# Acid-base variables in acute and chronic form of nontuberculous mycobacterial infection in growing goats experimentally inoculated with *Mycobacterium avium subsp*. *hominissuis* or *Mycobacterium avium* subsp. *paratuberculosis*

**DOI:** 10.1371/journal.pone.0243892

**Published:** 2020-12-14

**Authors:** Stefanie Bassis, Sina Fischer, Heike Köhler, Petra Reinhold

**Affiliations:** 1 Institute of Molecular Pathogenesis at ‘Friedrich-Loeffler-Institut’ (Federal Research Institute for Animal Health), Jena, Germany; 2 National Reference Laboratory for Paratuberculosis, Jena, Germany; University of British Columbia, CANADA

## Abstract

In current literature, data assessing the acid-base equilibrium in animals and humans during bacterial infection are rare. This study aimed to evaluate acid-base deteriorations in growing goats with experimentally induced NTM (nontuberculous mycobacteria) infections by application of the traditional Henderson-Hasselbalch approach and the strong ion model. NTM-challenged animals were orally inoculated with either *Mycobacterium avium* subsp. *hominissuis* (MAH; n = 18) or *Mycobacterium avium* subsp. *paratuberculosis* (MAP; n = 48). Twenty-five goats served as non-infected controls. Until 51^st^ week post-inoculation (wpi), blood gas analysis, serum biochemical analysis, and serum electrophoresis were performed on venous blood. Fifty percent (9/18) of goats inoculated with MAH developed acute clinical signs like apathy, fever, and diarrhea. Those animals died or had to be euthanized within 11 weeks post-inoculation. This acute form of NTM-infection was characterized by significantly lower concentrations of sodium, calcium, albumin, and total protein, as well as significantly higher concentrations of gamma globulin, associated with reduced albumin/globulin ratio. Acid-base status indicated alkalosis, but normal base excess and HCO_3_^-^ concentrations, besides significantly reduced levels of SID (strong ion difference), A_tot Alb_ (total plasma concentration of weak non-volatile acids, based on albumin), A_tot TP_ (A_tot_ based on total protein) and markedly lower SIG (strong ion gap). The remaining fifty percent (9/18) of MAH-infected goats and all goats challenged with MAP survived and presented a more sub-clinical, chronic form of infection mainly characterized by changes in serum protein profiles. With the progression of the disease, concentrations of gamma globulin, and total protein increased while albumin remained lower compared to controls. Consequently, significantly reduced albumin/globulin ratio and lower A_tot Alb_ as well as higher A_tot TP_ were observed. Changes were fully compensated with no effect on blood pH. Only the strong ion variables differentiated alterations in acid-base equilibrium during acute and chronic NTM-infection.

## Introduction

Any bacterial infection is a complex event challenging homeostasis in the host organism in diverse ways depending on the infection site, the pathogen, the pathology, and the severity of infection. Basic data assessing the general effects of bacterial infection on acid-base balance are rare. In experimental veterinary medicine, an acute respiratory acidosis and strong ion (metabolic) acidosis was documented in pigs with an induced respiratory *Chlamydia suis* infection [1]. A mixed interplay between respiratory alkalosis (due to hyperventilation) and counterbalancing metabolic effects were reported in calves inoculated with the respiratory pathogen *Chlamydia psittaci* [2]. The effect of subclinical bacterial infection on acid-base equilibrium has yet to be elucidated. In particular, the effect of NTM-infections on acid-base homeostasis has not been assessed in animals or men so far.

Two major approaches to evaluate acid-base status are currently available, i.e. the traditional calculations of pH, bicarbonate (HCO_3_^-^), base excess (BE), and anion gap (AG) based on the Henderson-Hasselbalch equation, and the more recent strong ion models [3–5]. With traditional values, four primary disorders can be detected: metabolic acidosis, metabolic alkalosis, respiratory acidosis, and respiratory alkalosis [5]. Mixed and complex acid-base disorders cannot be assessed [6]. The linear relationship between pCO_2_ and pH indicated by Henderson-Hasselbalch is criticized as non-correct, and HCO_3_^-^ cannot be regarded as an independent value of the metabolic component [4, 5]. Also, the temperature dependence of pH [7], and the dependence of the dissociation constant of carbonic acid (pK1’) on pH, temperature, protein concentration, and sodium concentration are not considered [4, 8, 9]. BE is criticized as non-accurate as it only shows a cumulative acid or base load and mixed acid-base disturbance may balance out [6, 10, 11].

In 1983, Stewart introduced the strong ion model based on the simultaneously valid principles of charge balance, dissociation equilibrium, and mass balance [3]. Thereby Stewart proposed that pH, H^+^, HCO_3_^-^, weak acids, and their acid residues are dependent variables, and are determined by three independent factors: (1) the strong ion difference (SID; strong cations minus strong anions); (2) acid total (A_tot_; total concentration of non-volatile weak acids: i.e. mainly albumin, globulin, and inorganic phosphate); and (3) pCO_2_ [3]. Constable simplified the strong ion model calculation methods, first provided methods for calculation of A_tot_ [4], and established an equation for calculating the strong ion gap (SIG) to determine unidentified anions in plasma [12]. The strong ion models allow to distinguish metabolic disorders in SID acidosis, SID alkalosis, A_tot_ acidosis, A_tot_ alkalosis and to record the presence of unexplained anions via SIG [5]. The calculated dependent and independent variables seem questionable from a chemical point of view and are criticized to provide no further benefit [13, 14]. Despite the critique, only the strong ion models can detect a complex, mixed acid-base disorder and consider the effect of electrolytes, phosphate, and buffering effect of proteins [3, 5, 6, 10, 15]. Recent research proposes the necessity to apply both methods for correct acid-base assessment [16].

This study aimed to provide a profound assessment of pathophysiological changes in acid-base balance associated with experimentally induced NTM-infections in a goat trial. This was to be achieved by longitudinal monitoring of traditional and strong ion variables, blood pH, serum proteins, electrolytes, and metabolites. We assumed changes in electrolytes, serum proteins, metabolites, and acid-base balance in goats with NTM-infections compared to healthy controls. These were to be characterized in detail. We hypothesized that the associated changes in acid-base balance could not fully be explained by the traditional values alone and that the strong ion variables were essential for correct interpretation.

## Materials and methods

### Legislation and ethical approval

Two consecutive animal experiments, each lasting 15 months (performed in the years 2011–2012 and 2013–2014) provided the experimental basis for this study. Animal experiments were carried out in strict accordance with European and National Law for the Care and Use of Animals. Protocols were approved by the Animal Health and Welfare Unit of the ‘Thüringer Landesamt für Verbraucherschutz’ (permit numbers: 04-001/11 and 04-002/12; dates of permission: 03.03.2011 and 12.12.2012, respectively). Experiments were done under the supervision of the authorized institutional Animal Protection Officer. During the entire study, every effort was made to minimize suffering.

### Study design and animals

Goat kids (from one farm with no history of mycobacterial infections) were admitted to the Federal Research Institute for Animal Health (Friedrich-Loeffler-Institut, FLI) in Jena at the age of 7 to 19 days, weighing 3.2 to 8.0 kg (5.6 ± 1.0 kg; mean ± SD). Upon arrival, fecal samples were collected and cultures from all animals were confirmed mycobacteria negative [17, 18].

For inoculation, two nontuberculous mycobacteria (NTM), *Mycobacterium avium* subsp. *paratuberculosis* (MAP) and *Mycobacterium avium* subsp. *hominissuis* (MAH), were chosen.

**Fig 1:**
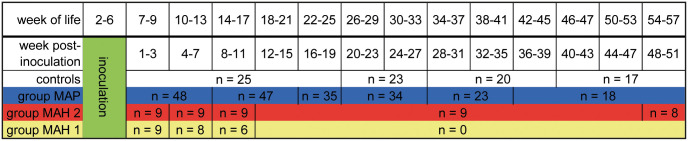
Study design. MAP, group infected with *Mycobacterium avium* subsp. *paratuberculosis*. MAH, group infected with *Mycobacterium avium* subsp. *hominissuis*.

Animals were randomly assigned to challenge groups and non-infected control groups as shown in Table 1. The groups were kept separately under biosecurity level 2 conditions. Challenges with MAP or MAH, respectively, started one week after the entrance to the premises. Goat kids to be inoculated were exposed to the pathogen orally via milk replacer. Each goat was challenged 10 times with intervals of 2–3 days between two challenges, leading to an inoculation period of 4 weeks (Fig 1). Controls received pure milk-replacer.

**Table 1:** Challenge groups and non-infected control groups of both trials.

	challenge with MAP (n = 48)	challenge with MAH (n = 21)	non-infected controls, CG (n = 25)
number of animals per trial	27 (04-001/11)	-	15 (04-001/11)
	21 (04-002/12)	21(04-002/12)	10 (04-002/12)
sex	34 M / 4 F	16 M / 3 F / 1 FI	23 M / 2 F
age at inoculation in days (mean ± SD)	39 ± 3.2	41 ± 2.5	41 ± 2.6
excluded from study	0	3	0
total inoculation dosage per animal	2.6 x 10^8^ cfu	2.13 x 10^10^ cfu	-
characteristics of bacterial strains	field isolate (JII-1961), non-pigmented type II strain [19]	isolate 09MA1289 from swine lymph node [18]	

MAP, *Mycobacterium avium* subsp. *paratuberculosis*. MAH, *Mycobacterium avium* subsp. *hominissuis*. CG, control group. M, male. F, female. FI, Female infertile. cfu, colony-forming units.

Each animal trial lasted from the 1^st^ to the 51^st^ week post-inoculation (wpi), which was equivalent to the 7^th^ to 57^th^ week of life (wl) (Fig 1). Clinical examinations were performed daily (rectally measured body temperature (BT), general behavior, appetite, consistency of feces, nasal and ocular discharge, presence or absence of cough, respiratory rate). Jugular venous blood was collected in 4-week intervals, about three hours after morning feeding. A heparinized 2 mL plastic PICO 50 syringe (Radiometer, Copenhagen, Denmark) and a 7.5 mL plastic Monovette syringe (AG & Co. KG, Sarstedt, Germany) per animal were filled anaerobically. Necropsy and pathological examination at defined time points were obligatory, resulting in a constantly decreasing number of animals (Fig 1).

### Re-grouping of animals exposed to MAH

Unexpectedly, goat kids challenged with MAH (n = 21) presented two opposite courses of infection. Nine animals fell seriously ill (MAH 1). Two of them died and seven had to be euthanized due to humane endpoints within the first 11 weeks after inoculation. Nine others turned into a mild progression of infection and survived until the end of the trial (MAH 2). Based on clinical and pathological examination, animals were reassigned to sub-group MAH 1 (acute form of MAH infection) or sub-group MAH 2 (chronic form of MAH infection) [18, 20]. Three MAH inoculated goats (one died, one euthanized due to humane endpoint, one was euthanized according to plan) could not be categorized by pathological examination and were excluded from the presented study.

### Housing conditions and animal welfare

Animals were kept under standardized conditions in air-conditioned rooms (20 ± 3 °C, 63 ± 6% relative humidity) on deep straw bedding. In the herd of origin, goat kids were raised conventionally with their mothers and had been fed colostrum. In the animal facility of FLI, the feeding regime was continuously adjusted to age and nutritional physiology (S1 Table). Water and meadow hay were supplied ad libitum. Male goats were castrated (6^th^ to 8^th^ wl) according to good veterinary practice under midazolam/ketamine general anesthesia and local anesthesia with lidocaine (intramuscular injection of 0.4 mg midazolam + 4 mg ketamine/kg body weight; Midazolam, Hexal, Holzkirchen, Germany; Ketamin, Intervet, Unterschleißheim, Germany; local injection into spermatic cords of 2 ml lidocaine/animal, Miocain 2%, bela-pharm, Vechta, Germany). For analgesia, goats received an intramuscular injection of phenylbutazone directly after surgery (20 mg/kg, Phenylbutazon 20%, CP-Pharma, Burgdorf, Germany) and intramuscular injections of metamizole one and two days postoperative (20–40 mg/kg, Metamizol, WDT, Garbsen, Germany). Goats received treatment against endo- and ectoparasites, and vitamin B supplementation as described elsewhere [17]. Goats inoculated with MAH that developed fever and apathy were treated with an intramuscular injection of metamizole (20 mg/kg, Metamizol, WDT, Garbsen, Germany). According to ethical standards, goats with severe clinical signs (apathy up to somnolence, a decrease of BT below physiological values, no feed intake) were euthanized. Euthanasia and necropsy were conducted as described elsewhere [17].

## Analytical methods

### Analysis of blood gases and electrolytes

Heparinized blood samples were kept at room temperature and were analyzed within 10 min after collection using a combined blood-gas and electrolyte analyzer (ABL725 Series, Radiometer; Copenhagen, Denmark). The following variables were measured by the analyzer: venous pH (pH(v)), partial pressure of CO_2_ (pCO_2_(v)), as well as plasma concentrations of sodium ([Na^+^]), potassium ([K^+^]), calcium ([Ca^2+^]), and chloride ([Cl^-^]). Electrolytes were measured via ion-selective potentiometry. Concentrations of glucose ([Gluc]) and _L_-lactate ([_L_-Lac]) in plasma were measured using the same equipment with enzymatic electrodes (glucose oxidase, lactate oxidase). Values of partial pressures and pH were corrected for BT measured rectally before each blood collection. Each sample was analyzed in duplicate and results were averaged.

### Serum biochemical analysis

Blood samples were centrifuged, serum harvested, and stored at -20 °C until analysis. The concentrations of total protein ([TP]) and inorganic phosphate ([iP]) were measured spectrophotometrically with the biuret method and ammonium-molybdate, respectively. The concentrations of albumin ([Alb]) and globulins, as well as globulin spectra, were measured by capillary electrophoresis with the Capillary 2 (Sebia; Evry Cedex, France).

### Calculated acid-base variables

The following variables were calculated by the blood-gas and electrolyte analyzer using proprietary equations included in the software: blood pH and pCO_2_ corrected for the rectally measured BT of the animal (pH(v)_BT_, pCO_2_(v)_BT_), hematocrit (Hct), standard bicarbonate ([HCO_3_^-^(st)]), actual base excess ([BE]), and standard base excess ([BE_Ecf_]).

### Henderson-Hasselbalch approach and anion gap

Bicarbonate was calculated via the Henderson-Hasselbalch equation. For calculation, pH(v)_BT_, pCO_2_(v)_BT_, the assumed value for solubility of carbon dioxide, S = 0.037 at 37 °C [8], and the dissociation constant for carbon dioxide pK1’ of 6.120 [7] were used. The anion gap, displaying the amount of unmeasured anion concentration, was calculated [5]:

$$AG=({[Na}^{+}]+{[K}^{+}])-\;({[Cl}^{-}]+{[HCO}_{3}^{-}])$$

### SID and strong ion approach

Strong ion difference was calculated from 3, 4, or 5 strong ions measured (_m_) in plasma:

$${SID}_{m3}=({[Na}^{+}]+{[K}^{+}])-{[Cl}^{-}]$$

$${SID}_{m4}=({[Na}^{+}]+{[K}^{+}])-({[Cl}^{-}]+[{}_{L}\text{-}Lac])$$

$${SID}_{m5}=({[Na}^{+}]+{[K}^{+}]+{[Ca}^{2+}])-({[Cl}^{-}]+[{}_{L}\text{-}Lac])$$

For goats and other small ruminants no data about plasma buffer capacity or values for A_tot_, as well as the effective dissociation constant for plasma weak acids K_a_ or its negative logarithm pK_a_, have been published. Values for cattle and calves are available and have been previously applied to goats [21]. Accordingly, the values described for calves were used [22]:

$$K_{a}=(0.84\pm 0.41)\times 10^{-7};\;{pK}_{a}=7.08$$

$$A_{tot\;TP}=[TP](g/L)\times 0.343$$

$$A_{tot\;Alb}=[Alb](g/L)\times 0.622$$

The strong ion gap (SIG) was calculated according to Constable [5, 12] from [TP] in g/L and from [Alb] in g/L, based on temperature-corrected pH values:

$${SIG}_{TP}=\frac{A_{tot\;TP}}{\left(1+10^{\left(pKa-pH\right)}\right)}-AG=([TP]\times \frac{0.343}{\left(1+10^{\left(pKa-pH\right)}\right)})-AG$$

$${SIG}_{Alb}=\frac{A_{tot\;Alb}}{\left(1+10^{\left(pKa-pH\right)}\right)}-AG=([Alb]\times \frac{0.622}{\left(1+10^{\left(7.08-pH\right)}\right)})-AG$$

### Statistical analysis

Exploratory statistical evaluation revealed that no significant differences existed between the two consecutive animal trials concerning the courses of MAP-infection or in non-infected controls over time. Thus, data from all animals exposed to MAP and all non-infected controls were merged into the respective comparison groups. Since the clinical courses of MAH-infections differed significantly between acute and chronic, the two MAH-subgroups (MAH 1, MAH 2) were analyzed separately.

Data were explored using frequency distributions and illustrated via boxplots presenting medians and 25–75% percentiles (boxes), with outlier values, given as circles (°), and extreme values given as stars (*). A descriptive statistic was performed for the whole study period. Data were summarized using medians and ranges (minimum, maximum). Evaluation of data by the Shapiro-Wilk test and histogram revealed that data was not normally distributed.

Due to the study design and circumstances, the number of animals decreased over study time. Within a given time-interval, the number of goats differed between groups but was constant within a group. The non-parametric Mann-Whitney *U*-test (MWU, Wilcoxon rank-sum test) was used to identify significant differences between independent groups within a given time interval. Due to the resulting low numbers of animals at the end of the study, the Mann-Whitney *U*-test was performed until the 28^th^ wpi. Within each dependent group, the variance of blood values over time was tested with the non-parametric Friedman test for significance. Significant differences were subsequently confirmed by the Wilcoxon Ranked Sum post hoc test. The test was applied to goats of one group that lived from 1^st^-3^rd^ wpi until the 24^th^-27^th^ wpi, or until the 8^th^-11^th^ wpi in sub-group MAH 1.

All analyses were carried out using SPSS Statistics Version 19.0 (IBM Corporation), R-Statistics, and Microsoft Excel 2010 (Microsoft Corporation). P values < 0.05 were considered as statistically significant.

## Results

### Two clinical courses of mycobacterial infections

By the end of the inoculation period, increased BT values (39.6 °C up to 41.1 °C) were recorded in goats challenged with MAH (S2 Table). Furthermore, mild depression to apathy, and intermittently soft feces up to pasty to liquid diarrhea were noted. About 50% of the MAH inoculated animals died or had to be euthanized before the 11^th^ wpi (sub-group MAH 1). The remaining 9 goats inoculated with MAH presented milder symptoms and recovered clinically (by gaining physiological BT data) until 12^th^-15^th^ wpi (S2 Table). These 9 MAH-exposed goats stayed clinically unsuspicious until the end of the study (sub-group MAH 2).

Inoculation with MAP resulted in a more homogenous course of infection. No acute onset of illness was seen, and all MAP-challenged goats developed subclinical to mild clinical forms of infection.

### General changes in metabolites and acid-base equilibrium observed in all NTM-challenged groups

Within the 1^st^-3^rd^ wpi, significantly higher blood concentrations of glucose and inorganic phosphate (iP) compared to 4^th^-7^th^ wpi were observed in all goat kids irrespective of infection, and median concentrations of _L_-Lac were above 1 mmol/L in all groups of goat kids (S2 Table). Up to the 12^th^-15^th^ wpi (4^th^-5^th^ month of life), [Gluc], [_L_-Lac] and [iP] decreased with age and stayed in the same range afterward until the end of the study in all surviving goats (S2–S5 Tables). Up to the 4^th^-5^th^ month of life, median [TP] values in the blood increased to about ≥ 130% in all surviving groups of goats compared to data after inoculation and stayed in the same range afterward (S6 Table). Despite that increase in [TP], all NTM-challenged groups showed lower [Alb] compared to controls during this time (S6 Table). Within the first weeks after inoculation, all NTM-challenged goats had lower [TP] and significantly lower [Gamma glob] compared to controls (S6 Table). Depending on the infection group, serum proteins then developed differently onwards. Within the 1^st^-3^rd^ wpi, significantly lower SIG_Alb_, and SIG_TP_, and higher AG were observed in all NTM-challenged animals compared to controls (Table 2, S6 Table). Thereby, SIG_Alb_ -median values in NTM-challenged goats were 3 to 4 times lower compared to the median value in controls (Table 2).

**Table 2:** Calculated anion gap and strong ion gap calculated on basis of albumin (SIG_Alb_) assessed in venous blood.

			AG mEq/L		SIG_Alb_ mEq/L	
wpi	group	n	median (min/max)		median (min/max)	
1–3	CG	25	13.3 (8.9/16.5)	a	-0.78 (-5.20/2.93)	c
	MAP	48	14.4 (10.7/17.1)	a	-2.45 (-5.49/0.15)	b
	MAH 2	9	15.9 (14.8/18.8)	b	-3.45 (-6.90/-0.90)	a
	MAH 1	9	15.3 (13.3/17.1)	b	-2.50 (-6.20/-1.50)	ab
4–7	CG	25	13.6 (11.1/19.8)	n.s.	0.21 (-4.63/4.88)	b
	MAP	48	13.55 (9.7/18.5)		0.86 (-2.50/4.54)	b
	MAH 2	9	14.6 (12.4/18.5)		-2.80 (-5.30/-0.50)	a
	MAH 1	8	13.9 (9.6/15.1)		-2.95 (-7.20/-1.80)	a
8–11	CG	25	16.5 (10.9/18.6)	b	-0.10 (-4.28/4.50)	b
	MAP	47	15.3 (7.2/19.8)	bc	0.30 (-3.50/7.20)	b
	MAH 2	9	16.9 (14.1/18.8)	bc	-2.50 (-7.70/-0.90)	a
	MAH 1	6	7.8 (6.8/13.8)	a	-3.90 (-10.40/-1.20)	a
12–15	CG	25	16.5 (13.6/21.9)	a	-0.50 (-6.00/3.43)	b
	MAP	47	18.4 (9.6/23.2)	ab	-1.70 (-8.90/4.51)	b
	MAH 2	9	20.0 (17.1/21.1)	b	-4.10 (-6.40/-2.4)	a
16–19	CG	25	15.9 (13.4/21.8)	a	0.40 (-7.70/2.52)	b
	MAP	35	16.2 (12.7/21.6)	a	-1.22 (-6.90/2.78)	b
	MAH 2	9	19.0 (16.6/21.3)	b	-3.90 (-5.40/-1.20)	a
20–23	CG	23	16.6 (11.1/21.1)	n.s.	0.16 (-6.80/4.60)	n.s.
	MAP	34	15.9 (13.3/19.8)		-1.21 (-5.60/4.70)	
	MAH 2	9	18.4 (8.6/20.1)		-2.30 (-4.50/8.30)	
24–27	CG	23	16.4 (14.1/18.5)	n.s.	0.24 (-2.76/4.50)	n.s.
	MAP	34	15.9 (13.4/18.4)		-0.30 (-2.80/3.30)	
	MAH 2	9	16.4 (13.1/18.8)		0.20 (-2.70/2.80)	
28–31	CG	20	15.0 (12.9/16.7)		1.26 (-0.71/5.70)	
	MAP	23	15.8 (13.7/18.8)		-0.20 (-3.11/3.40)	
	MAH 2	9	14.8 (12.7/16.9)		1.90 (0.90/3.40)	
32–35	CG	20	15.6 (12.4/17.1)		0.80 (-1.64/4.40)	
	MAP	23	15.7 (12.8/17.3)		0.56 (-2.49/3.60)	
	MAH 2	9	15.5 (14.1/16.7)		1.80 (0.10/4.00)	
36–39	CG	15	15.2 (12.9/17.0)		0.78 (-2.97/2.90)	
	MAP	18	15.5 (13.9/17.8)		-0.52 (-2.73/3.00)	
	MAH 2	9	14.4 (13.5/15.7)		2.10 (-0.30/2.50)	
40–43	CG	17	15.4 (13.3/18.4)		0.97 (-2.70/2.98)	
	MAP	17	15.7 (14.6/18.2)		-0.74 (-4.50/1.57)	
	MAH 2	9	17.4 (16.2/18.6)		-0.80 (-2.70/1.20)	
44–47	CG	17	15.4 (12.9/19.0)		1.40 (-3.20/3.45)	
	MAP	17	16.3 (13.7/17.7)		-0.50 (-3.60/2.64)	
	MAH 2	9	16.6 (15.1/18.9)		-0.10 (-3.00/2.10)	
48–51	CG	17	16.0 (14.3/19.4)		-0.40 (-2.51/3.70)	
	MAP	18	15.5 (12.6/17.8)		0.55 (-2.90/2.96)	
	MAH 2	8	16.5 (14.9/18.3)		-0.35 (-1.90/4.20)	

wpi, week post-inoculation. CG, control group. MAP, group infected with *Mycobacterium avium* subsp. *paratuberculosis*. MAH 1, sub-group infected with *Mycobacterium avium* subsp. *hominissuis* with acute, severe form of infection. MAH 2, sub-group with chronic form of infection. Different letters indicate significant differences between groups within one period (Mann-Whitney *U*-test, P < 0.05). n.s., no significant differences between groups in the given period. From 28^th^ week onwards Mann-Whitney *U*-test was not performed due to reduced numbers of observations. Significant differences within groups (Friedman test, P < 0.05) from 1^st^-3^rd^ to 24^th^-27^th^ wpi are given in S3–S5, and S10 Tables.

**Fig 2:**
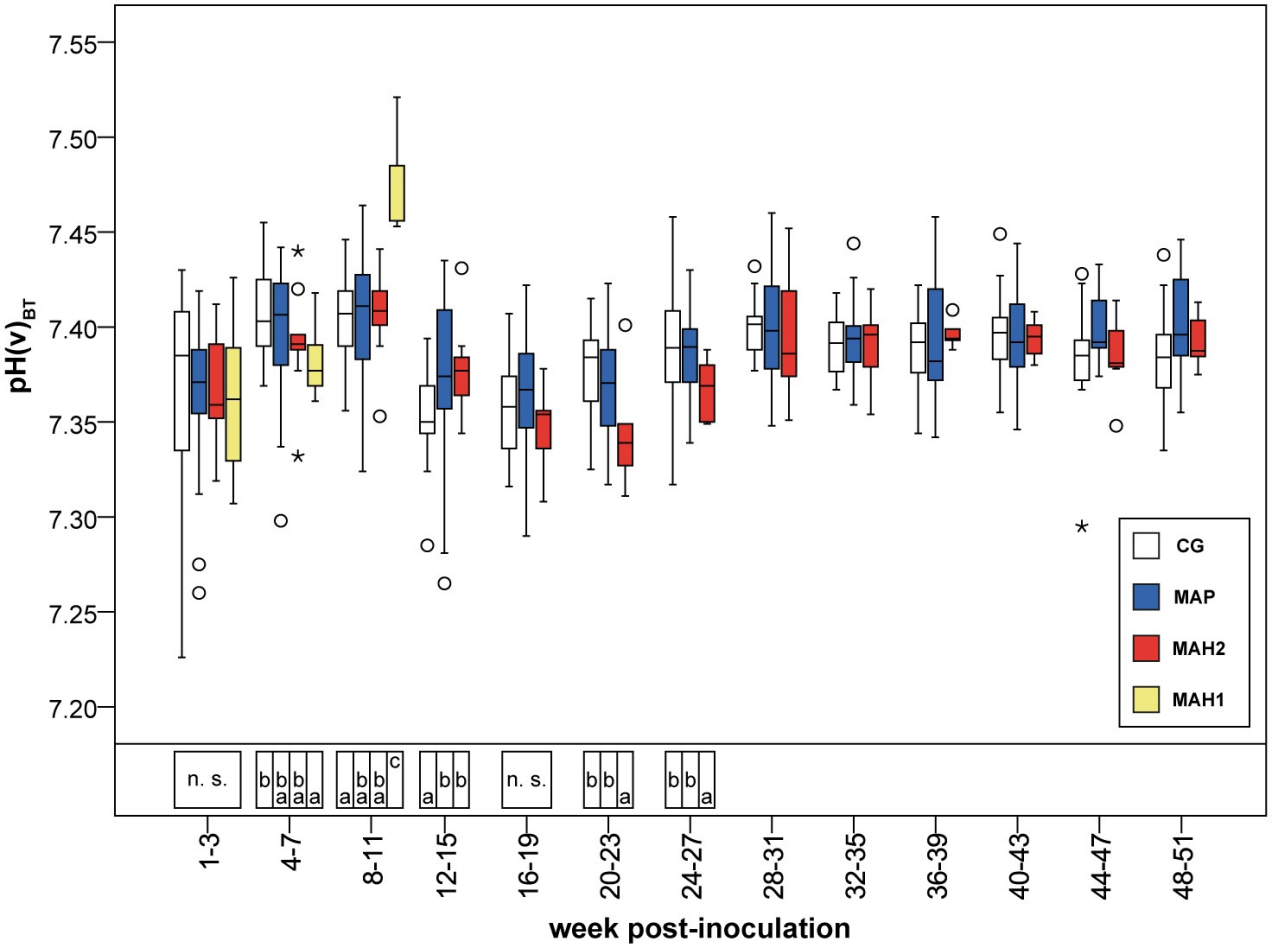
Measured pH values assessed in venous blood, corrected for actual body temperature. CG, control group. MAP, group infected with *Mycobacterium avium* subsp. *paratuberculosis*. MAH 1, sub-group infected with *Mycobacterium avium* subsp. *hominissuis* with acute, severe form of infection. MAH 2, sub-group with chronic form of infection. BT, body temperature (rectally measured before each blood collection). Different letters indicate significant differences between groups within one period (Mann-Whitney *U*-test, P < 0.05). n.s., no significant differences between groups in the given period. From 28^th^ week onwards Mann-Whitney *U*-test was not performed due to reduced numbers of observations. Significant differences within groups (Friedman test, P < 0.05) from 1^st^-3^rd^ to 24^th^-27^th^ wpi are given in S3–S5, and S10 Tables.

**Fig 3:**
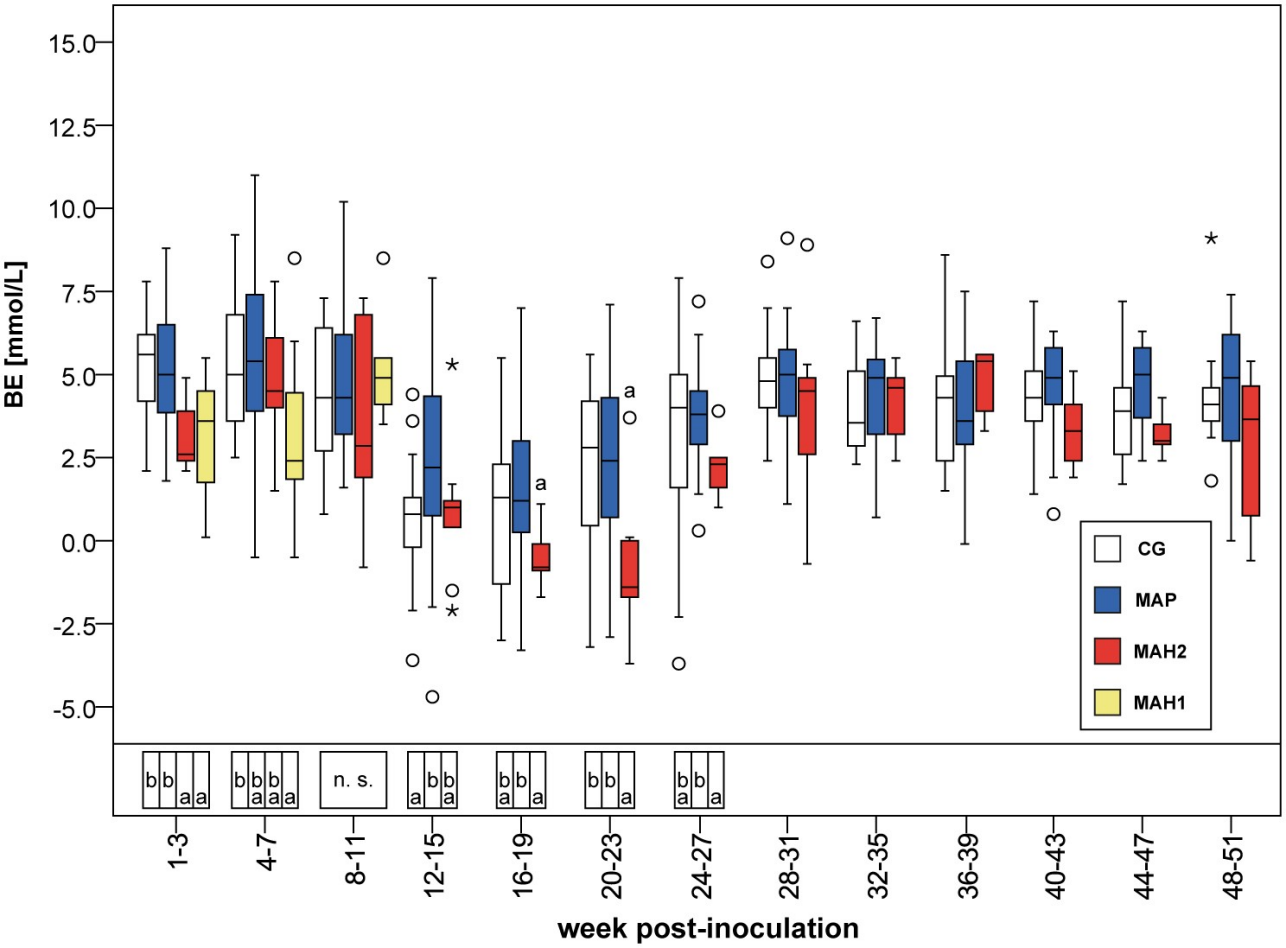
Calculated concentrations of base excess assessed in venous blood. CG, control group. MAP, group infected with *Mycobacterium avium* subsp. *paratuberculosis*. MAH 1, sub-group infected with *Mycobacterium avium* subsp. *hominissuis* with acute, severe form of infection. MAH 2, sub-group with chronic form of infection. Different letters indicate significant differences between groups within one period (Mann-Whitney *U*-test, P < 0.05). n.s., no significant differences between groups in the given period. From 28^th^ week onwards Mann-Whitney *U*-test was not performed due to reduced numbers of observations. Significant differences within groups (Friedman test, P < 0.05) from 1^st^-3^rd^ to 24^th^-27^th^ wpi are given in S3–S5, and S10 Tables.

After the observed increase in [TP], i.e. from the 12^th^-15^th^ wpi to the 20^th^-23^rd^ wpi (4–7 months of age), all remaining goats had a mild acidosis characterized by lower blood pH, significantly lower [HCO_3_^-^], [BE], [HCO_3_^-^(st)] and [BE_Ecf_], compared to values within 3 weeks after inoculation (Table 3, Figs 2 and 3, S3–S6 Tables).

**Table 3:** Concentration of calculated bicarbonate and measured partial CO_2_ pressure (corrected for body temperature) assessed in venous blood.

			pCO_2(V)BT_ kPa		[HCO_3_^-^] mmol/L	
wpi	group	n	median (min/max)		median (min/max)	
1–3	CG	25	7.31 (6.30/10.70)	bc	30.54 (27.01/32.26)	b
	MAP	48	7.32 (6.33/10.10)	c	30.47 (26.98/33.81)	b
	MAH 2	9	6.86 (6.23/7.74)	ab	27.75 (26.96/29.48)	a
	MAH 1	9	6.70 (6.26/8.52)	a	28.08 (24.90/30.18)	a
4–7	CG	25	6.67 (5.63/7.50)	n.s.	29.35 (26.32/33.38)	b
	MAP	48	6.68 (5.88/9.90)		29.63 (23.64/35.68)	ab
	MAH 2	9	6.67 (5.95/8.03)		29.82 (25.40/31.42)	ab
	MAH 1	8	6.41 (5.86/7.64)		26.94 (23.56/33.22)	a
8–11	CG	25	6.49 (5.72/7.32)	c	28.37 (25.46/31.83)	n.s.
	MAP	47	6.43 (5.25/7.74)	bc	28.58 (24.46/34.54)	
	MAH 2	9	5.99 (5.81/6.93)	abc	26.40 (23.51/31.48)	
	MAH 1	6	5.52 (4.44/5.86)	a	26.26 (25.29/31.27)	
12–15	CG	25	6.42 (5.89/7.10)	b	25.34 (22.05/28.78)	a
	MAP	47	6.43 (5.59/7.51)	b	26.47 (21.51/31.77)	b
	MAH 2	9	5.94 (5.19/6.76)	a	24.87 (21.55/28.75)	ab
16–19	CG	25	6.43 (5.67/6.91)	n.s.	25.87 (21.52/29.61)	ab
	MAP	35	6.46 (5.55/7.52)		25.71 (22.14/31.40)	b
	MAH 2	9	6.12 (5.82/6.66)		23.67 (23.29/25.19)	a
20–23	CG	23	6.47 (5.49/7.03)	ab	27.42 (21.39/29.52)	b
	MAP	34	6.54 (5.64/7.52)	b	26.98 (21.50/31.00)	b
	MAH 2	9	6.24 (5.79/6.88)	a	23.20 (21.09/27.57)	a
24–27	CG	23	6.35 (5.48/7.18)	a	27.45 (21.17/31.09)	ab
	MAP	34	6.63 (5.73/7.46)	b	28.12 (25.32/31.10)	b
	MAH 2	9	6.61 (5.79/7.14)	ab	26.54 (24.71/28.90)	a
28–31	CG	20	6.62 (5.95/7.25)		28.89 (26.41/32.57)	
	MAP	23	6.63 (5.69/7.49)		29.16 (25.42/33.45)	
	MAH 2	9	6.42 (5.72/7.45)		28.43 (23.31/32.44)	
32–35	CG	20	6.65 (6.07/7.07)		28.01 (26.15/31.03)	
	MAP	23	6.68 (5.83/7.33)		29.23 (25.29/31.05)	
	MAH 2	9	6.55 (6.02/7.86)		28.62 (26.35/31.02)	
36–39	CG	15	6.64 (5.94/7.15)		28.50 (26.24/32.96)	
	MAP	18	6.62 (5.92/7.17)		28.32 (24.39/30.78)	
	MAH 2	5	6.63 (6.43/6.92)		29.64 (27.44/29.95)	
40–43	CG	17	6.61 (6.03/7.01)		28.45 (26.19/31.33)	
	MAP	17	6.62 (6.05/7.07)		29.07 (25.23/30.31)	
	MAH 2	9	6.21 (5.83/6.84)		27.19 (25.81/29.09)	
44–47	CG	17	6.63 (5.92/8.31)		28.34 (25.74/31.03)	
	MAP	17	6.55 (5.95/7.20)		29.13 (26.82/30.87)	
	MAH 2	9	6.49 (6.09/7.30)		27.26 (26.61/28.42)	
48–51	CG	17	6.75 (6.21/7.69)		28.47 (26.46/33.19)	
	MAP	18	6.55 (5.89/7.06)		28.49 (24.18/31.51)	
	MAH 2	8	6.20 (5.40/6.89)		27.69 (22.94/29.54)	

wpi, week post-inoculation. CG, control group. MAP, group infected with *Mycobacterium avium* subsp. *paratuberculosis*. MAH 1, sub-group infected with *Mycobacterium avium* subsp. *hominissuis* with acute, severe form of infection. MAH 2, sub-group with chronic form of infection. BT, body temperature (rectally measured before each blood collection). Different letters indicate significant differences between groups within one period (Mann-Whitney *U*-test, P < 0.05). n.s., no significant differences between groups in the given period. From 28^th^ week onwards Mann-Whitney *U*-test was not performed due to reduced numbers of observations. Significant differences within groups (Friedman test, P < 0.05) from 1^st^-3^rd^ to 24^th^-27^th^ wpi are given in S3–S5, and S10 Tables.

In all groups [Cl^-^] ranged from 94 to 111 mmol/L, and [Na^+^] from 134 to 150 mmol/L throughout the study (S7 Table). Besides these fluctuating concentrations significantly higher [Cl^-^] were present in all NTM-challenged animals within the 1^st^-3^rd^ and the 4^th^-7^th^ wpi (S7 Table). Simultaneously [Na^+^] tended to be higher compared to controls (S7 Table).

### Changes in acid-base equilibrium associated with MAH inoculation

By the end of inoculation, during 1^st^-3^rd^ wpi, all MAH-challenged animals showed significantly lower [HCO_3_^-^], [HCO_3_^-^(st)], [BE], [BE_Ecf_], SIG_TP_, and significantly higher AG values, (but normal blood pH) compared to group MAP and controls (Tables 2 and 3, Figs 2 and 3, S6 and S8 Tables). While clinical signs in sub-group MAH 1 and MAH 2 were already present (S2 Table) during this time, no significant differences in acid-base variables, electrolytes, and metabolites were obvious. Only concentrations of beta 2 globulin in MAH 2 were significantly higher than in MAH 1 (S9 Table).

**Fig 4:**
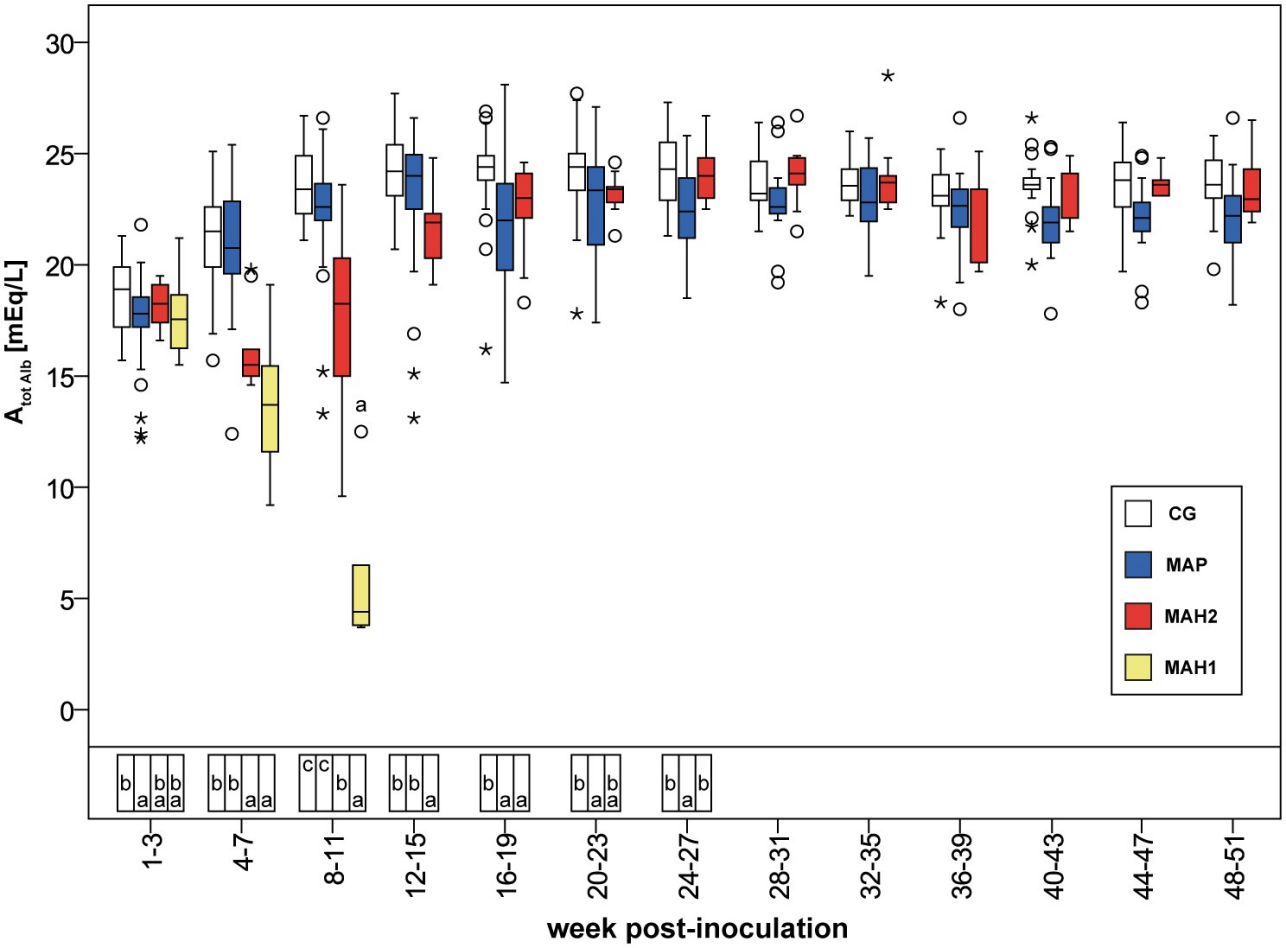
Calculated acid total based on albumin in mEq/L assessed in venous blood. CG, control group. MAP, group infected with *Mycobacterium avium* subsp. *paratuberculosis*. MAH 1, sub-group infected with *Mycobacterium avium* subsp. *hominissuis* with acute, severe form of infection. MAH 2, sub-group with chronic form of infection. Different letters indicate significant differences between groups within one period (Mann-Whitney *U*-test, P < 0.05). n.s., no significant differences between groups in the given period. From 28^th^ week onwards Mann-Whitney *U*-test was not performed due to reduced numbers of observations. Significant differences within groups (Friedman test, P < 0.05) from 1^st^-3^rd^ to 24^th^-27^th^ wpi are given in S3–S5, and S10 Tables.

From the 1^st^-3^rd^ wpi to the 4^th^-7^th^ wpi median [Gamma glob] significantly increased to 320% (sub-group MAH 2) and 422% (sub-group MAH 1). Simultaneously, median [Alb] significantly decreased to 87% (sub-group MAH 2) and 80% (sub-group MAH 1) (S5, S6 and S10 Tables). In sub-group MAH 1, this decrease in [Alb] was not significant. Thereby in both MAH sub-groups, changes in [Gamma glob] and [Alb] balanced out to [TP] values that were comparable to values in controls, and no differences within the 4^th^-7^th^ wpi in SIG_TP_ values compared to the other groups were observed (S6 Table). Regarding [Alb] also A_tot Alb_ decreased significantly in all MAH-challenged goats (Fig 4, S6 Table). [Alb], A_tot Alb_, and SIG_Alb_ remained significantly lower in both MAH sub-groups until the 8^th^-11^th^ wpi, compared to group MAP and controls (Table 2, Fig 4 and S6 Table).

Overall, these changes in protein pattern in MAH-exposed goats were reflected by a significant decrease of the albumin/globulin ratio from the 1^st^-3^rd^ to the 8^th^-11^th^ wpi. This decrease to only 21.2% was significantly more pronounced in sub-group MAH 1 compared to sub-group MAH 2 (to 56.5%) (Table 4, S5 and S10 Tables).

**Table 4:** Albumin/Globulin ratio, acid total based on total protein (A_tot TP_) assessed in venous blood.

			Alb/Glob ratio		A_tot TP_ mEq/L	
wpi	group	n	median (min/max)		median (min/max)	
1–3	CG	25	1.48 (0.97/1.87)	n.s.	17.5 (15.5/19.9)	c
	MAP	48	1.52 (0.63/2.08)		16.5 (15.0/18.5)	a
	MAH 2	9	1.38 (1.11/1.66)		17.2 (16.6/18.0)	bc
	MAH 1	9	1.60 (1.19/1.79)		16.5 (14.4/18.2)	abc
4–7	CG	25	1.34 (0.78/1.76)	c	20.4 (16.1/25.0)	b
	MAP	48	1.54 (0.45/2.38)	d	19.3 (16.7/23.0)	a
	MAH 2	9	0.85 (0.58/1.39)	b	18.9 (17.8/22.4)	ab
	MAH 1	8	0.57 (0.37/0.87)	a	21.0 (15.8/26.8)	ab
8–11	CG	25	1.41 (1.08/1.63)	c	22.3 (18.7/25.1)	ab
	MAP	47	1.48 (0.39/1.98)	c	21.1 (18.0/26.3)	c
	MAH 2	9	0.78 (0.56/1.39)	b	22.4 (18.6/24.6)	bc
	MAH 1	6	0.34 (0.24/0.45)	a	11.5 (9.6/23.4)	a
12–15	CG	25	1.40 (1.05/1.72)	b	22.8 (19.5/26.3)	b
	MAP	47	1.41 (0.62/1.99)	b	22.0 (17.0/25.1)	a
	MAH 2	9	1.07 (0.77/1.42)	a	23.2 (21.6/25.4)	ab
16–19	CG	25	1.41 (0.84/1.72)	b	23.0 (19.5/26.0)	b
	MAP	35	1.28 (0.60/1.92)	a	22.2 (19.4/26.4)	a
	MAH 2	9	1.17 (0.74/1.47)	a	22.6 (21.5/25.6)	ab
20–23	CG	23	1.40 (0.89/1.67)	b	22.8 (20.5/25.1)	n.s.
	MAP	34	1.24 (0.78/1.75)	a	23.2 (19.5/24.9)	
	MAH 2	9	1.17 (0.92/1.43)	a	23.3 (21.0/26.2)	
24–27	CG	23	1.40 (1.22/1.66)	b	22.4 (20.8/25.9)	a
	MAP	34	1.21 (0.86/1.70)	a	22.7 (21.0/25.3)	a
	MAH 2	9	1.36 (0.92/1.47)	ab	24.0 (21.5/25.9)	b
28–31	CG	20	1.40 (1.25/1.62)		22.2 (20.2/24.6)	
	MAP	23	1.21 (0.84/1.67)		22.8 (20.6/24.2)	
	MAH 2	9	1.37 (0.94/1.45)		23.2 (22.0/26.7)	
32–35	CG	20	1.36 (1.12/1.63)		22.7 (21.4/24.7)	
	MAP	23	1.28 (0.84/1.73)		22.6 (21.3/24.3)	
	MAH 2	9	1.38 (0.89/1.46)		23.0 (21.6/26.8)	
36–39	CG	15	1.42 (1.03/1.53)		22.6 (17.3/23.5)	
	MAP	18	1.26 (0.78/1.57)		22.7 (21.0/24.0)	
	MAH 2	9	1.07 (1.03/1.37)		22.4 (21.0/24.9)	
40–43	CG	17	1.43 (1.26/1.78)		21.9 (19.8/24.0)	
	MAP	17	1.20 (0.67/1.43)		22.3 (20.8/27.9)	
	MAH 2	9	1.31 (0.98/1.49)		22.8 (21.5/24.0)	
44–47	CG	17	1.44 (1.22/1.65)		22.2 (19.8/23.8)	
	MAP	17	1.24 (0.67/1.51)		22.8 (20.6/25.8)	
	MAH 2	9	1.33 (1.04/1.53)		23.2 (21.3/25.0)	
48–51	CG	17	1.41 (1.16/1.67)		22.1 (19.7/25.2)	
	MAP	18	1.11 (0.69/1.44)		23.3 (21.1/26.4)	
	MAH 2	8	1.26 (0.78/1.49)		23.2 (21.8/27.7)	

wpi, week post-inoculation. CG, control group. MAP, group infected with *Mycobacterium avium* subsp. *paratuberculosis*. MAH 1, sub-group infected with *Mycobacterium avium* subsp. *hominissuis* with acute, severe form of infection. MAH 2, sub-group with chronic form of infection. Different letters indicate significant differences between groups within one period (Mann-Whitney *U*-test, P < 0.05). n.s., no significant differences between groups in the given period. From 28^th^ week onwards Mann-Whitney *U*-test was not performed due to reduced numbers of observations. Significant differences within groups (Friedman test, P < 0.05) from 1^st^-3^rd^ to 24^th^-27^th^ wpi are given in S3–S5, and S10 Tables.

#### The manifestation of an acute severe form

**Fig 5:**
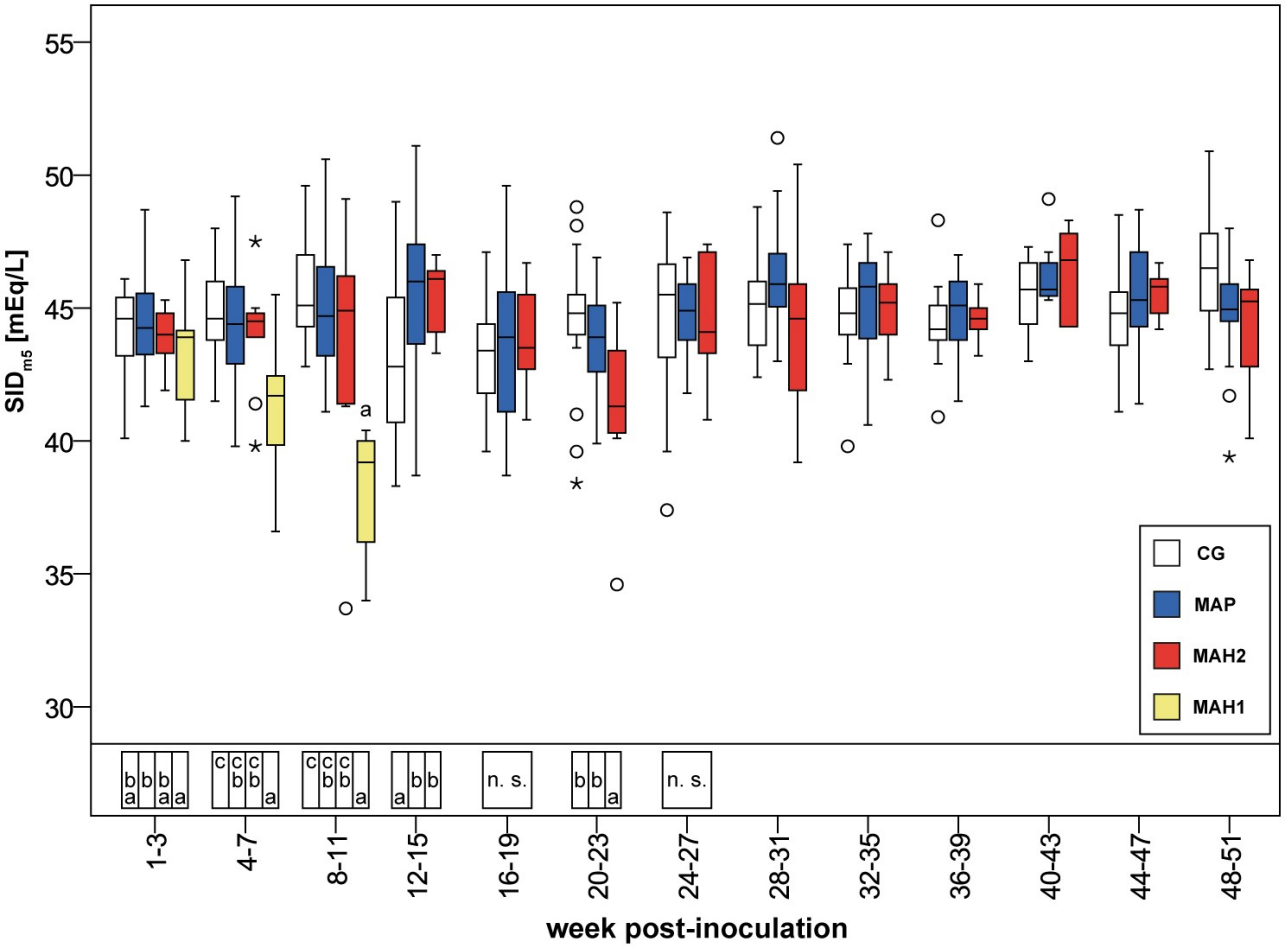
Calculated strong ion difference based on 5 measured strong ions assessed in venous blood. CG, control group. MAP, group infected with *Mycobacterium avium* subsp. *paratuberculosis*. MAH 1, sub-group infected with *Mycobacterium avium* subsp. *hominissuis* with acute, severe form of infection. MAH 2, sub-group with chronic form of infection. Different letters indicate significant differences between groups within one period (Mann-Whitney *U*-test, P < 0.05). n.s., no significant differences between groups in the given period. From 28^th^ week onwards Mann-Whitney *U*-test was not performed due to reduced numbers of observations. Significant differences within groups (Friedman test, P < 0.05) from 1^st^-3^rd^ to 24^th^-27^th^ wpi are given in S3–S5, and S10 Tables.

The observed increase of [Gamma glob] from the 1^st^-3^rd^ to the 4^th^-7^th^ wpi in goats inoculated with MAH was significantly higher in sub-group MAH 1 compared to sub-group MAH 2 (S6 Table). From the 1^st^-3^rd^ to the 4^th^-7^th^ wpi [HCO_3_^-^] and [BE] or [HCO_3_^-^(st)] and [BE_Ecf_], respectively, decreased in sub-group MAH 1 (Table 3, Fig 3, and S8 Table). Simultaneously SID_m3_, SID_m4_, and SID_m5_ decreased by about 5–6% in sub-group MAH 1, leading to significantly lower SID-concentrations compared to all other groups (Table 5, Fig 5). Despite that, [Cl^-^] median values in sub-group MAH 1 increased from 105 mmol/L to 107 mmol/L, while no difference in [Na^+^] was obvious between all groups (S7 Table). pCO_2_(v)_BT_ and pH(v)_BT_ did not change significantly (Table 3, Fig 2). The decreases in [HCO_3_^-^], [BE], [HCO_3_^-^(st)], and [BE_Ecf_] as well as in SID_m3_, SID_m4_, and SID_m5_ were not statistically significant (S10 Table).

**Table 5:** Calculated strong ion difference based on 3 and 4 strong ions assessed in venous blood.

			SID_m3_ mEq/L		SID_m4_ mEq/L	
wpi	group	n	median (min/max)		median (min/max)	
1–3	CG	25	44.4 (39.3/46.9)	ab	43.2 (38.8/44.8)	ab
	MAP	48	44.6 (41.4/49.2)	b	42.9 (40.0/47.4)	b
	MAH 2	9	44.4 (42.2/45.8)	ab	42.9 (40.6/43.9)	ab
	MAH 1	9	43.3 (39.4/46.5)	a	42.5 (38.5/45.4)	a
4–7	CG	25	44.2 (41.0/48.4)	c	43.3 (40.2/46.8)	c
	MAP	48	43.8 (39.5/49.0)	bc	43.1 (38.5/47.9)	bc
	MAH 2	9	43.8 (39.0/48.5)	bc	43.4 (38.5/46.2)	bc
	MAH 1	8	40.7 (36.6/44.6)	a	40.2 (35.3/44.2)	a
8–11	CG	25	44.7 (42.0/49.6)	c	43.9 (41.4/48.4)	c
	MAP	47	44.5 (40.4/50.6)	bc	43.5 (39.8/49.3)	bc
	MAH 2	9	44.8 (40.6/48.5)	bc	43.8 (40.0/47.8)	bc
	MAH 1	6	37.6 (33.1/40.2)	a	36.7 (32.6/39.4)	a
12–15	CG	25	42.4 (37.3/48.6)	a	41.5 (36.9/47.8)	a
	MAP	47	45.3 (37.8/50.3)	b	44.6 (37.4/49.8)	b
	MAH 2	9	45.2 (42.5/46.2)	b	44.8 (42.0/45.6)	b
16–19	CG	25	42.4 (39.0/47.6)	n.s.	42.0 (38.4/45.9)	n.s.
	MAP	35	43.2 (37.8/48.7)		42.6 (37.4/48.3)	
	MAH 2	9	42.6 (39.9/45.9)		42.2 (39.5/45.4)	
20–23	CG	23	43.9 (37.6/47.8)	b	43.6 (37.1/47.5)	b
	MAP	34	43.2 (39.0/46.1)	b	42.6 (38.5/45.6)	b
	MAH 2	9	40.5 (33.7/44.7)	a	40.0 (33.2/43.9)	a
24–27	CG	23	44.8 (36.5/48.2)	n.s.	44.2 (36.1/47.4)	n.s.
	MAP	34	44.1 (41.3/46.1)		43.6 (40.5/45.6)	
	MAH 2	9	43.2 (39.9/47.3)		42.8 (39.5/46.1)	
28–31	CG	20	44.5 (41.4/48.1)		43.9 (41.1/47.5)	
	MAP	23	45.2 (42.1/50.6)		44.7 (41.7/50.1)	
	MAH 2	9	44.0 (38.3/49.7)		43.4 (37.9/49.3)	
32–35	CG	20	44.1 (38.9/46.8)		43.6 (38.6/46.2)	
	MAP	23	45.0 (39.8/47.0)		44.6 (39.3/46.6)	
	MAH 2	9	44.8 (41.4/46.7)		44.0 (41.0/45.8)	
36–39	CG	15	43.5 (40.0/47.6)		42.9 (39.7/47.1)	
	MAP	18	44.5 (40.7/46.8)		43.8 (40.2/45.8)	
	MAH 2	9	43.8 (42.4/45.1)		43.4 (42.0/44.7)	
40–43	CG	17	45.1 (42.3/46.8)		44.5 (41.8/46.1)	
	MAP	17	45.2 (44.7/48.5)		44.5 (44.1/47.9)	
	MAH 2	9	45.9 (43.5/47.5)		45.6 (43.1/47.1)	
44–47	CG	17	44.1 (40.2/47.6)		43.6 (39.8/47.2)	
	MAP	17	44.8 (41.4/47.8)		44.0 (40.2/47.5)	
	MAH 2	9	44.8 (43.4/47.5)		44.5 (43.0/45.5)	
48–51	CG	17	45.7 (41.7/49.9)		45.2 (41.3/49.6)	
	MAP	18	44.4 (38.3/47.7)		43.7 (38.1/46.7)	
	MAH 2	8	44.4 (39.7/45.8)		44.0 (39/45.5)	

wpi, week post-inoculation. CG, control group. MAP, group infected with *Mycobacterium avium* subsp. *paratuberculosis*. MAH 1, sub-group infected with *Mycobacterium avium* subsp. *hominissuis* with acute, severe form of infection. MAH 2, sub-group with chronic form of infection. Different letters indicate significant differences between groups within one period (Mann-Whitney *U*-test, P < 0.05). n.s., no significant differences between groups in the given period. From 28^th^ week onwards Mann-Whitney *U*-test was not performed due to reduced numbers of observations. Significant differences within groups (Friedman test, P < 0.05) from 1^st^-3^rd^ to 24^th^-27^th^ wpi are given in S3–S5, and S10 Tables.

#### Failure of homeostasis in sub-group MAH 1

From the 4^th^-7^th^ wpi to the 8^th^-11^th^ wpi the acute severe form of MAH-infection proceeded and all animals of sub-group MAH 1 died or needed to be euthanized (Fig 1). Thereby in MAH 1, blood pH increased significantly (Fig 2, S10 Table) while [TP], [Alb], A_tot Alb_, A_tot TP_, AG, [Na^+^], [Ca^2+^], [Gluc] decreased significantly (Fig 4, Tables 2 and 4, S2, S6, S7 and S10 Tables). Besides these changes, no significant differences were observed within the 8^th^-11^th^ wpi in [HCO_3_^-^] and [BE] or [HCO_3_^-^(st)], and [BE_Ecf_], respectively, comparing sub-group MAH 1 to the other groups (Table 3, Fig 3, S8 Table). Median values of [Alb] decreased by 61.4% to 30.8% of median values measured within 3 weeks after inoculation. [Gamma glob] decreased by 35% and [TP] by 45.2% (S6 Table). Consequently, also median values of A_tot Alb_ and A_tot TP_ decreased (Table 4, Fig 4). Within 8^th^-11^th^ wpi significantly lower [Gluc] median values of 2.9 mmol/L, as well as significantly lower [TP] and [Alb] compared to all other groups were measured (S2 and S6 Tables). Thereby, the lowest individually measured [TP] of 28.0 g/dL and [Alb] of 6.0 g/dL within this study were noted (S6 Table). Median values of pCO_2_(v)_BT_ significantly decreased in both MAH sub-groups from the 4^th^-7^th^ wpi to the 8^th^-11^th^ wpi (S5 and S10 Tables). In sub-group MAH 1 pCO_2_(v)_BT_ decreased by 14% and in sub-group MAH 2 pCO_2_(v)_BT_ by 10% (Table 3). While [Cl^-^] did not change, significant decrease in [Na^+^] median values by about 4.2% to 138 mmol/L and [Ca^2+^] median values by about 18.5% to 1.06 mmol/L (S7 Table) led to a drop in SID_m3_, SID_m4_, and SID_m5_. Consequently, significantly lower median values of 37.6 mEq/L SID_m3_, 36.7 mEq/L SID_m4_, and 37.7 mEq/L SID_m5_ in sub-group MAH 1 compared to all other groups were observed (Table 5, Fig 5).

#### Recovery of sub-group MAH 2

From the 1^st^-3^rd^ wpi to the 4^th^-7^th^ wpi, [HCO_3_^-^], [BE], and pCO_2_(v)_BT_ as well as [HCO_3_^-^(st)], and [BE_Ecf_] increased in sub-group MAH 2 to values similar to group MAP and to control group (Table 3, Fig 3, S8 Table). From the 4^th^-7^th^ wpi to the 8^th^-11^th^ wpi median values of [Alb] increased by 20.3% and [Gamma glob] median values increased significantly by 55.2% in sub-group MAH 2 (S5 and S6 Tables). Consequently, A_totAlb_ also increased. Thereby, observed median values of [Alb] (30.2 g/dL) were significantly lower while median values of [Gamma glob] (19.4 g/dL) were significantly higher compared to group MAP and to control group. After the 8^th^-11^th^ wpi, [Alb] continued to increase in sub-group MAH 2, and [Gamma glob] decreased until values matched concentrations in MAP-inoculated goats from the 20^th^-23^rd^ wpi onwards (S6 Table).

The mild acidosis observed in all groups after the increase of [TP] lasted 4 weeks longer until the 24^th^-27^th^ wpi in sub-group MAH 2, and significantly lower [HCO_3_^-^], [BE], [HCO_3_^-^(st)], and [BE_Ecf_] were reached compared to group MAP and to control group (Table 3, Fig 3, and S8 Table).

### Changes in acid-base equilibrium associated with MAP inoculation

Throughout the study, [Alb] in goats exposed to MAP tended to be lower, and was occasionally significantly lower, compared to controls (S6 Table). Until 16^th^-19^th^ wpi [Gamma glob] and [TP] also tended to be lower and were occasionally significantly lower. From 20^th^-23^rd^ wpi onwards, i.e. with the progression of the disease, [TP] and [Gamma glob] were higher compared to controls (S6 Table).

By comparing group MAP with sub-group MAH 2, no significant differences in [Gamma glob] or [TP] were observed. Otherwise [Alb] tended to be lower and was occasionally significantly lower in group MAP, from the 16^th^-19^th^ wpi onwards. These changes were accompanied by a significantly lower albumin/globulin ratio (Table 4). Consequently, A_tot Alb_ tended to be lower while A_tot TP_ tended to be higher in MAP challenged goats compared to sub-group MAH 2 (Table 4, Fig 4).

## Discussion

This study provides essential information for (i) biomedical science using large animal models, (ii) comparative medicine concerning the host response to mycobacterial infections, (iii) translational medicine assessing pathophysiology of acute versus chronic bacterial infection, and (iv) veterinary medicine.

Two clinical outcomes after NTM-inoculation, a chronic and an acute form, both associated with changes in acid-base equilibrium, were the main findings of this study. Acute onset of disease after NTM-inoculation was characterized by significant deteriorations of acid-base variables as well as electrolytes and metabolites, altogether indicating a severe failure of homeostasis. Chronic and subclinical courses of NTM-infection, respectively, were associated with alterations in serum proteins and the strong ion variables related to albumin or total protein, (i.e. A_tot Alb_, A_tot TP_, SIG_Alb_, and SIG_TP_). Besides changes in the acid-base balance associated with infection, physiological changes due to somatic growth and development of rumination could not be excluded and were valid for all groups. The latter had previously been evaluated in detail taking only the non-infected controls into account [21].

### The animal model

MAP or MAH were used for inoculation. MAP is the causative agent of Johne’s disease, a chronic gastrointestinal disease in domestic and wild ruminants [23]. Johne’s disease is characterized by a long clinically inapparent phase followed by diarrhea and wasting as main clinical signs [23]. MAH is a ubiquitous pathogen, mainly causing mild to subclinical infections in swine and humans, like children or immunosuppressed persons [24–27]. The susceptibility of goats to MAH-infection has recently been documented experimentally by this interdisciplinary working group [18, 20]. While the dosage used for MAP-inoculation was based on previous studies [17], there was no experience regarding the dosage to be used for MAH-inoculation. Based on the idea that ubiquitous bacteria would present lower pathogenicity compared to MAP, a relatively high cumulative dosage of MAH (2.13 x 10^10^ cfu per goat; Table 1) was administered, and a subclinical to chronic course of disease in goats was assumed. The acute, severe form of illness that appeared in 50% of goats exposed to MAH, within the first few weeks after inoculation, was not expected. Nevertheless, severe MAH-infections with fever, diarrhea, emaciation, and gastro-intestinal granulomas leading to death or euthanasia have been reported occasionally in individual cats, dogs, and horses [28–30]. It is likely that the effects of inoculation dosage and infection pressure within the group contributed to the acute onset.

Although arterial blood-gas analysis is regarded as the gold standard in acid-base evaluation, central venous blood is also valuable to evaluate both metabolic and respiratory components of acid-base status [31, 32]. Following a previous study assessing acid-base status in young pigs [1], jugular venous blood samples were used in this study. The low number of animals within both MAH sub-groups and constantly declining numbers of animals within sub-group MAH 1 must be critically kept in mind during interpretation.

### General observed acid-base changes after NTM inoculation

Irrespective of clinical signs of infection, lower up to significantly lower [Alb], [Gamma glob] and [TP] were observed shortly after inoculation, and were interpreted as unspecific signs of inflammation. Reduced [Alb] levels in NTM-challenged goats were expected and can be linked to decreased formation due to the acute phase reaction [33]. Likewise, lower [Gamma glob] could be linked to early infection [33]. The acute phase response is induced by pro-inflammatory cytokines (interleukin 1, interleukin 6, TNF α) released by activated monocytes or after sustained tissue damage [34]. Previously a positive association between SIG and concentrations of inflammatory cytokines was demonstrated [35]. In accordance with these findings significantly more negative SIG_Alb_, SIG_TP_, and significantly higher AG values were observed in all NTM-inoculated animals during early infection. SIG_Alb_ remained significantly more negative during the clinically critical time in MAH-exposed animals, indicating ongoing inflammation and tissue damage. Increased levels of the pro-inflammatory cytokine IFNγ (interferon-gamma) in blood serum of MAH-exposed goats support this postulated association between SIG and the level of inflammation (S1 Fig). Overall, the impact of acute phase reaction and inflammation processes on acid-base homeostasis appears unspecific and does not allow conclusions about localization or origin.

### Inter-individual differences in host-pathogen interaction, leading to an acute form of NTM-infection

The different clinical outcomes of MAH-exposure in a homogenous group of animals (same breed, age, farm of origin, and randomized allocation to groups) indicated inter-individual differences regarding the host pathogen-immune interaction. An inter-individual variation in host immune response to mycobacteria is known from human and bovine tuberculosis [18]. The histopathological examination of all MAH-exposed goats revealed similarities with human and bovine tuberculosis [18, 20].

In goats with the acute severe form of illness histopathologic examination demonstrated an exuberant inflammatory response and associated severe tissue damage [20]. Furthermore, 6 goats showed signs consistent with multi-organ dysfunction syndrome (MODS) and systemic inflammatory response syndrome (SIRS) (i.e., multifocal, renal necrosis; multiple fibrin thrombi in the kidneys, the liver, and the lungs) [18]. Although biochemical parameters were not evaluated, clinical signs, histopathology, measured metabolites (significantly lower [Gluc], and [Alb]), and acid-base variables indicated sepsis before death or euthanasia. Sepsis is defined as a dysregulated systemic inflammatory and immune response to bacterial infection leading to life-threatening organ dysfunctions [36, 37].

Overall, the results of histopathologic examination [18, 20] and the significant increases in gamma globulins [33] indicated a greater host-pathogen immune interaction in the acute form of NTM-infection.

### Changes in the acid-base balance associated with an acute form of NTM-infection

After inoculation, hypocapnia and significantly lower [HCO_3_^-^] and [BE], or [HCO_3_^-^(st)] and [BE_Ecf_], respectively, were observed within both MAH sub-groups compared to group MAP and to control group. The loss of pCO_2_(v)_BT_ was most likely induced by hyperventilation caused by fever reactions observed in most of the MAH-inoculated goats. Since blood pH(v)_BT_ was not affected, those changes were fully compensated. Despite the clinical presence of diarrhea in some goats inoculated with NTM, elevated blood [Cl^-^] and [Na^+^] as well as normal hematocrit excluded any diarrhea-induced acidotic burden on the group level. Consequently, traditional acid-base variables were consistent with compensated respiratory alkalosis. Applying the strong ion approach, normal SID values, and lower A_tot_ values also indicated a compensated primary respiratory disorder.

Although blood pH(v)_BT_ was significantly lowered in acute severely ill goats 4^th^-7^th^ wpi, the animals were still able to maintain it within the physiological range. Traditional variables, i.e., significantly lower [HCO_3_^-^], [HCO_3_^-^(st)], [BE], and [BE_Ecf_] as well as normal pCO_2_(v)_BT_ compared to controls, revealed a metabolic acidotic burden, with no conclusion about origin [5]. This is in good agreement with results found in man, associating low serum bicarbonate with higher mortality, independent of systemic pH(v)_BT_ values [38], and the known correlation between a lower [BE] and worse outcome in critical illness [39, 40]. The strong ion variables indicated a SID acidosis or a SIG acidosis, respectively, balanced out by an A_tot_ alkalosis. Significantly lower SID revealed changes in electrolytes as causal factors for the metabolic acidotic burden (regardless of the number of electrolytes incorporated in the calculation) [3]. This seemed to be a cumulative effect, as it was not expected when looked at electrolytes separately. Decreasing albumin levels led to an A_tot_ alkalosis [41, 42]. According to the strong ion approach, a decrease in plasma SID can be caused by intestinal loss of cations due to diarrhea [6], and some diarrhea was observed in acutely ill goats. Furthermore, it is proposed that low SID correlates with hypoalbuminemia to maintain electro-neutrality and acid-base equilibrium [43]. Whether declining albumin levels contributed to lower SID values could not be determined.

Recent investigations have demonstrated that interactions do exist between acid-base disorders and the underlying inflammation process in critical illness [44–49]. Therefore it is proposed that acidosis alters the release of inflammatory mediators, plays a role in the progression of an illness, and the pathogenesis of sepsis [50]. This agrees with the course of the constantly proceeding acute, severe form of NTM-infection, leading to failure of acid-base homeostasis, and assumed sepsis during 8^th^-11^th^ wpi. During sepsis, a variety of related acid-base disorders are known: primary respiratory alkalosis, various forms of primary metabolic acidosis, complex acid-base disorders, the appearance of unexplained anions, and lactate acidosis [36, 51, 52]. In the present study, traditional parameters like hypocapnia, unaffected bicarbonate, and base excess indicated an acute respiratory alkalosis during 8^th^-11^th^ wpi [5]. Thereby, traditional variables failed to reveal the complexity of the acid-base disorders present. The strong ion variables revealed a SID acidosis, and a SIG_Alb_ acidosis overwhelmed by a massive A_tot_ alkalosis (A_tot Alb_ as well as A_tot TP_) beside a mild respiratory alkalosis. This is supported by findings demonstrating severe acid-base disturbances despite presenting normal BE in every sixth patient hospitalized at Intensive Care Units [11]. As the BE only shows a cumulative acid or base load, mixed acid-base disturbance may balance out [6, 10, 11]. The fact that [HCO_3_^-^] was not affected during this complex acid-base disorders confirms that bicarbonate cannot be regarded as an independent variable for the metabolic component [4, 5]. Significantly lower SID_m3_, SID_m4_, and SID_m5_ values were mainly caused by dramatically low [Na^+^] and [Ca^2+^]. This may have been caused by gastrointestinal and renal loss of electrolytes and loss of sodium via effusions [53–55]. Histopathologic examination in acutely ill goats showed massive renal and gastrointestinal defects, thoracic and abdominal effusions [18, 20]. Furthermore, assumed sepsis in acutely ill goats may have contributed to low SID values. It is known that inflammatory cytokines induce a lower parathyroid hormone (PTH) secretion as well as a PTH resistance in kidneys and bones during sepsis [56]. Albumin is a weak acid [11, 41, 42], and the dramatic drop in all protein concentrations in all severely ill goats before death or euthanasia caused a massive alkalinizing effect. Hypoalbuminemia is a well-known complication in critical illness that is associated with a poor outcome in humans [57–59]. Hypoalbuminemia may have been caused by reduced production within the liver [33], due to acute-phase reaction [34], or may have been associated with liver failure that was indicated by histological examination [18]. Furthermore, a loss of albumin via kidneys or the gastrointestinal tract, gastrointestinal malabsorption, as well as reduced feed intake [33] due to apathy, may have worsened hypoproteinemia.

### Chronic NTM-infection and associated acid-base changes

Nine MAH-inoculated goats and all animals exposed to MAP evolved a chronic form of infection. With ongoing chronicity of infection (20^th^-23^rd^ wpi onwards), SIG values decreased indicating a lower intensity of inflammation [35]. Simultaneously, significantly higher [Gamma glob] in blood indicated stimulation of antibody production [33]. Despite similarities in SIG and [Gamma glob], [Alb] developed differently in goats with chronic NTM-infection over time: [Alb] in sub-group MAH 2 tended to reach the level of controls while concentrations of albumin remained low in goats exposed to MAP until the end of the study. On the one hand, this may underline ongoing recovery in sub-group MAH 2. On the other hand, this possibly shows the progression of disease in group MAP. These differences were only reflected in strong ion variables A_tot Alb_ and A_tot TP_, without any effect on other acid-base variables.

### Methodological aspects

The strong ion variables A_tot_ and SIG are not only species-specific [4, 12] but also highly dependent on serum protein pattern as both values can be calculated based on either albumin or total protein [4]. In all animals exposed to MAH, SIG_TP_ and SIG_Alb_ values developed reversely in parallel to a constantly declining albumin/globulin ratio. Both SIG_TP_, as well as AG, were not able to detect unmeasured anions correctly in MAH exposed goats during hypoalbuminemia. Hypoalbuminemia is known to cause inaccuracies in AG [60, 61]. Furthermore, A_tot TP_ only changed at the time when homeostasis in sub-group MAH 1 failed, while significantly lower A_tot Alb_ was already obvious 4 weeks before. Therefore, under conditions of variable albumin/globulin ratios (especially during infection), SIG and A_tot_ should be calculated on basis of albumin as well as on basis of total protein and should be interpreted together. Moreover, in critical illness, SIG_Alb_ might be preferably used instead of SIG_TP_, as albumin carries the main base-binding capacity of the non-volatile weak acids [62].

The correct determination of SID is dependent on precise electrolyte measurement, and values differ depending on measuring methods and instruments [4, 63]. The effect of hypoalbuminemia and acid-base disturbances on ionization of electrolytes, and consequently on correct measurements, is still unclear. Regarding ionized Ca^2+^, concentrations are mainly dependent on albumin concentration and blood pH [54, 64]. Lower [Ca^2+^] in sub-group MAH 1 during failure of homeostasis could therefore partly be due to the measuring procedure. It is known that alkalosis is associated with hypopotassemia and hyperchloremia [54, 65]. Despite that, no difference in [Cl^-^] or [K^+^] in sub-group MAH 1 during the critically ill phase, and alkalemia were obvious. For correct interpretation of SID during critical illness, further research is needed to better identify the effects of hypoproteinemia and pH changes on measurements of electrolytes.

SID values showed high potentials in early detection of a derailment of acid-base equilibrium. Overall, SID can be calculated easily based on measured electrolytes and seems a readily available tool.

## Conclusions

This study provides new essential information about the long-term processes of NTM-infections, and the resulting consequences on acid-base equilibrium.

There is strong evidence for an association between SIG and the level of inflammation during bacterial infection. SIG seems a promising additional parameter to detect inflammation, making further research on this topic worthwhile.

Acute NTM-infection led to substantial imbalances in homeostasis, accompanied by massive hypoalbuminemia, significantly lower A_tot_ and SID values, hypocapnia, alkalosis, and signs of SIRS or MODS, respectively, consistent with sepsis. Significantly decreased SID, base excess, and bicarbonate were observed before the derailment of homeostasis in acute NTM-infection.

Chronic NTM-infection was dominated by alterations of blood protein profiles, mainly characterized by low concentrations of Albumin, higher gamma globulin, and thereby lower A_tot Alb_.

The present results demonstrate that the effects of acute and chronic bacterial infection and critical illness on acid-base equilibrium can only be understood by considering the strong ion variables.

## Supporting information

S1 Fig Levels of the pro-inflammatory cytokine IFNγ (interferon-gamma) assessed in blood serum of goats. CG, control group. MAP, group infected with *Mycobacterium avium* subsp. *paratuberculosis*. MAH 2, group infected with *Mycobacterium avium* subsp. *hominissuis* with chronic form of infection.
(TIF)

S1 Table Feeding regime. (PDF)

S2 Table Concentrations of _L_-Lactate, glucose, inorganic phosphate in mmol/L assessed in venous blood and rectally measured body temperature. wpi, week post-inoculation. CG, control group. MAP, group infected with *Mycobacterium avium* subsp. *paratuberculosis*. MAH 1, sub-group infected with *Mycobacterium avium* subsp. *hominissuis* with acute, severe form of infection. MAH 2, sub-group with chronic form of infection. Different letters indicate significant differences between groups within one period (Mann-Whitney *U*-test, P < 0.05). n.s., no significant differences between groups in the given period. From 28^th^ week onwards Mann-Whitney *U*-test was not performed due to reduced numbers of observations. Significant differences within groups (Friedman test, P < 0.05) from 1^st^-3^rd^ to 24^th^-27^th^ wpi are given in S3–S5, and S10 Tables.
(PDF)

S3 Table P-values of Friedman test and consequently followed post hoc Wilcoxon rank-sum test applied to controls (CG) from the 1^st^-3^rd^ to the 24^th^-27^th^ week post-inoculation (wpi). Additional information to S3 Table: P-values > 0.05 were considered not significant.
(PDF)

S4 Table P-values of Friedman test and consequently followed post hoc Wilcoxon rank-sum test applied to group MAP from the 1^st^-3^rd^ to the 24^th^-27^th^ week post-inoculation (wpi). Additional information to S4 Table: P-values > 0.05 were considered not significant.
(PDF)

S5 Table P-values of Friedman test and consequently followed post hoc Wilcoxon rank-sum test applied to sub-group MAH 2 from the 1^st^-3^rd^ to the 24^th^-27^th^ week post-inoculation (wpi). Additional information to S5 Table: P-values > 0.05 were considered not significant.
(PDF)

S6 Table Concentrations of total protein, albumin, gamma globulin in g/dL, and strong ion gap calculated on basis of total protein (SIG_TP_) in mEq/L assessed in venous blood. wpi, week post-inoculation. CG, control group. MAP, group infected with *Mycobacterium avium* subsp. *paratuberculosis*. MAH 1, sub-group infected with *Mycobacterium avium* subsp. *hominissuis* with acute, severe form of infection. MAH 2, sub-group with chronic form of infection. Different letters indicate significant differences between groups within one period (Mann-Whitney *U*-test, P < 0.05). n.s., no significant differences between groups in the given period. From 28^th^ week onwards Mann-Whitney *U*-test was not performed due to reduced numbers of observations. Significant differences within groups (Friedman test, P < 0.05) from 1^st^-3^rd^ to 24^th^-27^th^ wpi are given in S3–S5, and S10 Tables.
(PDF)

S7 Table Concentrations of sodium, chloride, potassium, and calcium in mmol/L assessed in venous blood. wpi, week post-inoculation. CG, control group. MAP, group infected with *Mycobacterium avium* subsp. *paratuberculosis*. MAH 1, sub-group infected with *Mycobacterium avium* subsp. *hominissuis* with acute, severe form of infection. MAH 2, sub-group with chronic form of infection. Significant differences P < 0.05 calculated via Mann-Whitney U-test, a: CG/MAP, b: CG/MAH 2, c: CG/MAH 1, d: MAP/MAH 2, e: MAP/MAH 1, f: MAH 1/MAH 2. Significant differences P < 0.05 calculated via Friedman test from 1^st^-3^rd^ to 24^th^-27^th^ wpi, ^1^: within group CG, ^2^: within group MAP, ^3^: within sub-group MAH 2, ^4^: from 1^st^-3^rd^ to 8^th^-11^th^ wpi within sub-group MAH 1. Detailed P-values are given in S3–S5 and S10 Tables.
(PDF)

S8 Table Concentrations of standard bicarbonate and standard base excess in mmol/L and hematocrit assessed in venous blood. wpi, week post-inoculation. CG, control group. MAP, group infected with *Mycobacterium avium* subsp. *paratuberculosis*. MAH 1, sub-group infected with *Mycobacterium avium* subsp. *hominissuis* with acute, severe form of infection. MAH 2, sub-group with chronic form of infection. Different letters indicate significant differences between groups within one period (Mann-Whitney *U*-test, P < 0.05). n.s., no significant differences between groups in the given period. From 28^th^ week onwards Mann-Whitney *U*-test was not performed due to reduced numbers of observations. Significant differences within groups (Friedman test, P < 0.05) from 1^st^-3^rd^ to 24^th^-27^th^ wpi are given in S3–S5, and S10 Tables.
(PDF)

S9 Table Concentrations of beta 1, beta 2, alpha 1, and alpha 2 globulin in g/dL assessed in venous blood. wpi, week post-inoculation. CG, control group. MAP, group infected with *Mycobacterium avium* subsp. *paratuberculosis*. MAH 1, sub-group infected with *Mycobacterium avium* subsp. *hominissuis* with acute, severe form of infection. MAH 2, sub-group with chronic form of infection. Different letters indicate significant differences between groups within one period (Mann-Whitney *U*-test, P < 0.05). n.s., no significant differences between groups in the given period. From 28^th^ week onwards Mann-Whitney *U*-test was not performed due to reduced numbers of observations. Significant differences within groups (Friedman test, P < 0.05) from 1^st^-3^rd^ to 24^th^-27^th^ wpi are given in S3–S5, and S10 Tables.
(PDF)

S10 Table P-values of Friedman test and consequently followed post hoc Wilcoxon rank-sum test applied to sub-group MAH 1 from the 1^st^-3^rd^ to the 8^th^-11^th^ week post-inoculation (wpi). Additional information to S10 Table: P-values > 0.05 were considered not significant.
(PDF)
