# Supplementary figures and images for: Acid-base variables in acute and chronic form of nontuberculous mycobacterial infection in growing goats experimentally inoculated with Mycobacterium avium subsp. hominissuis or Mycobacterium avium subsp. paratuberculosis
Source: PLoS One. 2020 Dec 14;15(12):e0243892. doi: 10.1371/journal.pone.0243892 (PMC7735625; doi:10.1371/journal.pone.0243892)

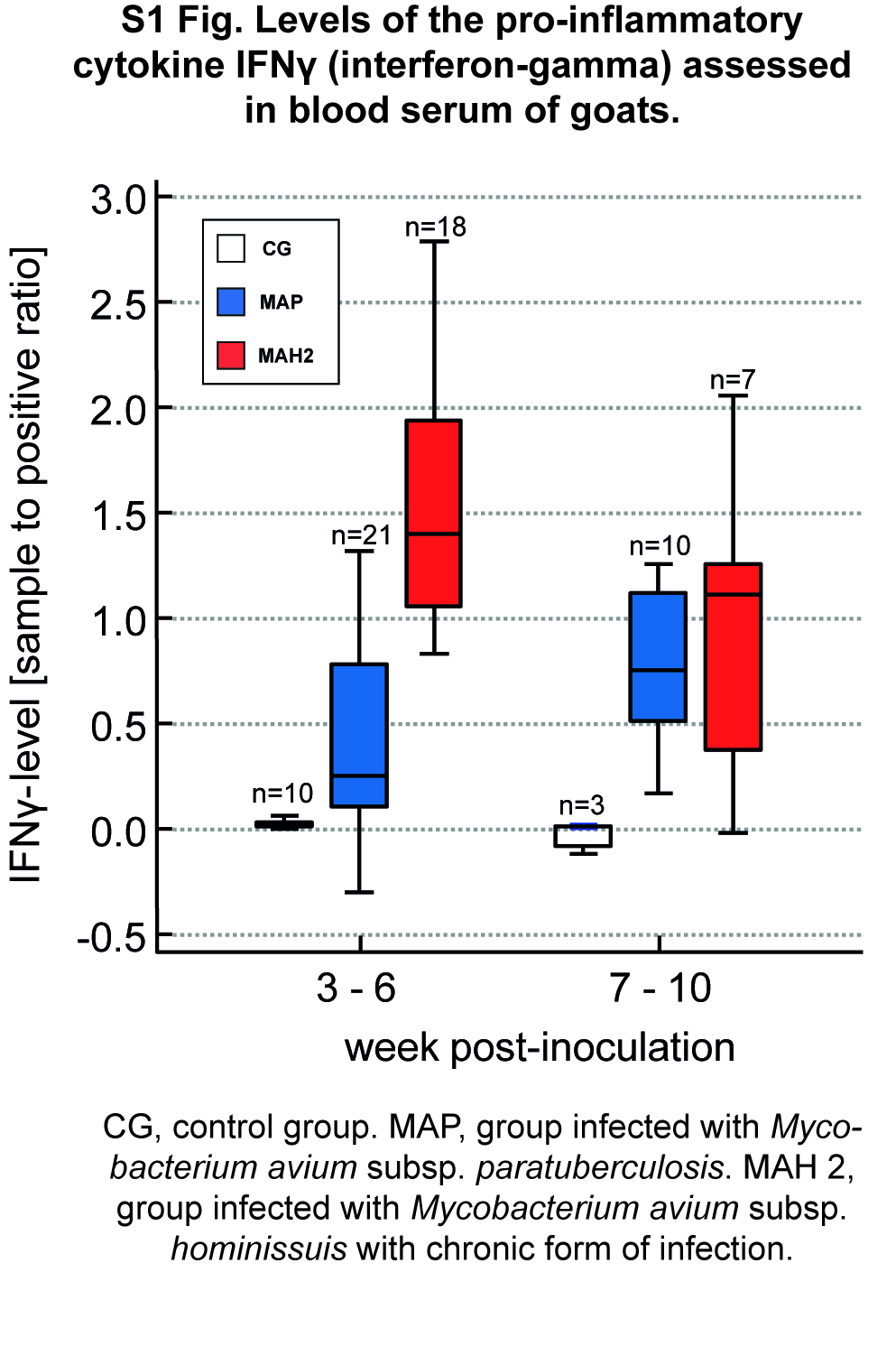

Supplement: S1 Fig — CG, control group. MAP, group infected with Mycobacterium avium subsp. paratuberculosis. MAH 2, group infected with Mycobacterium avium subsp. hominissuis with chronic form of infection. (TIF) [file pone.0243892.s001.tif]
