# Supplementary material for: Acid-base variables in acute and chronic form of nontuberculous mycobacterial infection in growing goats experimentally inoculated with Mycobacterium avium subsp. hominissuis or Mycobacterium avium subsp. paratuberculosis
Source: PLoS One. 2020 Dec 14;15(12):e0243892. doi: 10.1371/journal.pone.0243892 (PMC7735625; doi:10.1371/journal.pone.0243892)
Supplement: S1 Table — (PDF) [file pone.0243892.s002.pdf]

S1 Table: Feeding regime

age	feed				
**1st – 6th week of life**	colostrum, milk - raised conventionally with their mothers				
**6th – 51st week of life**	meadow hay ad libitum				
**age**	**feedstuff**	**manufacturer**	**content**		
**6th – 12th week of life**	milk replacer	Denkamilk Capri-Ovi (Germany)	analytical key constitutions	raw protein	21.5 %
				crude fat	24.2 %
				crude fiber	0.0 %
				crude ash	7.6 %
				phosphorus	0.63 %
			food additives per kg	copper sulfate	2 mg
				vitamin A	6.000 UI
				vitamin D3	2.000 UI
				vitamin E	150 UI
			composition	whey flour, vegetable oil (coconut, palm, rape), wheat protein hydrolyzed, wheat flour	
**6th – 12th week of life**	concentrates for goat kids	LHG Schmölln (Germany)	analytical key constitutions	raw protein	16.0 %
				crude fat	2.2 %
				crude fiber	6.1 %
				crude ash	7.0 %
				calcium	1.1 %
				phosphorus	0.5 %
				sodium	0.15 %
			food additives per kg	vitamin A	15.000 UI
				vitamin D3	1.500 UI
				vitamin E	13.0 mg
			composition	barley, wheat, soya beans extract meal, wheat bran, dried sugar beet plump, sugar beet molasses	
**6th – 51st week of life**	dairy concentrates for goats	LHG Schmölln (Germany)	analytical key constitutions	raw protein	18.0 %
				crude fat	2.5 %
				crude fiber	8.1 %
				crude ash	7.2 %
				calcium	1.0 %
				phosphorus	0.65 %
				sodium	0.25 %
			food additives per kg	vitamin A	7.000 UI
				vitamin D3	800 UI
				vitamin E	10.0 mg
				ion sulfate	20.0 mg
				zinc oxide	90.0 mg
				manganese oxide	20.0 mg
				calcium iodate	0.5 mg
				selenium	0.4 mg
				cobalt carbonate	0.3 mg
			composition	barley, rapeseed extraction meal, sunflower seed extraction meal, wheat bran, dried sugar beet plump, sugar beet molasses	
