# Supplementary material for: Acid-base variables in acute and chronic form of nontuberculous mycobacterial infection in growing goats experimentally inoculated with Mycobacterium avium subsp. hominissuis or Mycobacterium avium subsp. paratuberculosis
Source: PLoS One. 2020 Dec 14;15(12):e0243892. doi: 10.1371/journal.pone.0243892 (PMC7735625; doi:10.1371/journal.pone.0243892)
Supplement: S2 Table — wpi, week post-inoculation. CG, control group. MAP, group infected with Mycobacterium avium subsp. paratuberculosis. MAH 1, sub-group infected with Mycobacterium avium subsp. hominissuis with acute, severe form of infection. MAH 2, sub-group with chronic form of infection. Different letters indicate significant differences between groups within one period (Mann-Whitney U-test, P < 0.05). n.s., no significant differences between groups in the given period. From 28th week onwards Mann-Whitney U-test was not performed due to reduced numbers of observations. Significant differences within groups (Friedman test, P < 0.05) from 1st-3rd to 24th-27th wpi are given in S3–S5, and S10 Tables. (PDF) [file pone.0243892.s003.pdf]

S2 Table: Concentrations of L-lactate, glucose, inorganic phosphate in mmol/L assessed in venous blood and rectally measured body temperature.

			[L-Lac] mmol/L		[Gluc] mmol/L		[iP] mmol/L		body temperature °C	
**wpi**	**group**	**n**	**median (min/max)**		**median (min/max)**		**median (min/max)**		**median (min/max)**	
1-3	CG	25	1.2 (0.5/3.7)	a	5.2 (4.3/7.4)	a	3.15 (1.60/4.16)	a	39.3 (38.9/40.2)	ab
	MAP	48	1.7 (0.6/4.0)	b	5.8 (4.3/9.3)	b	3.59 (2.91/4.62)	c	39.2 (38.5/40.0)	a
	MAH 2	9	1.6 (0.6/2.7)	ab	5.5 (4.8/8.0)	ab	3.06 (2.44/3.68)	ab	39.5 (39.0/40.6)	bc
	MAH 1	9	1.1 (0.6/3.2)	ab	5.6 (4.2/6.5)	ab	3.13 (2.69/3.39)	ab	39.7 (39.3/40.5)	c
4-7	CG	25	0.8 (0.5/1.8)	c	4.9 (3.6/6.0)	c	2.86 (1.84/3.96)		39.1 (38.6/39.6)	a
	MAP	48	0.7 (0.2/3.1)	bc	4.7 (2.8/6.9)	bc	2.64 (1.52/3.99)	n.s.	39.2 (38.6/40.3)	ab
	MAH 2	9	0.4 (0.4/2.3)	a	4.0 (3.0/5.3)	ab	2.72 (1.61/3.29)		39.5 (39.3/40.9)	bc
	MAH 1	8	0.6 (0.3/1.6)	abc	3.4 (2.8/5.0)	a	2.42 (1.26/2.91)		40.3 (39.5/40.5)	c
8-11	CG	25	0.8 (0.4/1.5)		4.4 (3.8/5.2)	c	2.76 (1.52/3.60)	b	38.9 (38.4/39.7)	a
	MAP	47	0.8 (0.4/2.3)	n.s.	4.5 (3.5/5.0)	bc	2.54 (1.86/3.34)	ab	39.1 (38.3/39.4)	b
	MAH 2	9	0.8 (0.5/2.3)		4.2 (3.7/4.9)	abc	2.64 (1.95/2.97)	ab	39.0 (38.8/39.7)	c
	MAH 1	6	0.7 (0.5/0.9)		2.9 (1.7/4.0)	a	2.17 (1.64/2.87)	a	40.2 (39.2/41.1)	c
12-15	CG	25	0.5 (0.2/1.5)		3.9 (3.4/4.4)	ab	2.06 (1.12/2.95)		38.9 (38.5/39.9)	
	MAP	47	0.5 (0.2/3.2)	n.s.	3.9 (3.4/4.5)	a	2.16 (1.47/3.13)	n.s.	38.7 (38.1/40.2)	n.s.
	MAH 2	9	0.5 (0.3/0.8)		4.0 (3.8/4.3)	b	2.08 (1.29/3.04)		38.9 (38.4/39.4)	
16-19	CG	25	0.5 (0.2/1.7)		3.8 (3.4/4.4)		1.79 (1.09/2.47)		38.9 (38.5/39.7)	a
	MAP	35	0.5 (0.2/1.2)	n.s.	3.8 (3.3/4.3)	n.s.	1.96 (0.96/2.75)	n.s.	38.9 (38.1/40.8)	a
	MAH 2	9	0.5 (0.3/0.7)		3.8 (3.3/4.1)		1.70 (1.50/2.67)		39.2 (38.6/39.4)	b
20-23	CG	23	0.5 (0.2/0.9)	a	3.6 (2.8/4.8)		1.83 (1.26/2.27)		39.0 (38.5/40.2)	
	MAP	34	0.5 (0.3/1.1)	b	3.8 (2.8/6.0)	n.s.	1.92 (1.20/2.61)	n.s.	39.0 (38.4/39.7)	n.s.
	MAH 2	9	0.5 (0.4/0.8)	ab	3.6 (3.2/6.6)		2.00 (1.29/2.37)		38.8 (38.0/39.4)	
24-27	CG	23	0.4 (0.3/1.2)		3.6 (2.8/4.1)		1.89 (1.10/2.37)		38.8 (38.3/39.4)	ab
	MAP	34	0.5 (0.2/0.8)	n.s.	3.5 (2.2/4.3)	n.s.	1.95 (1.13/2.90)	n.s.	39.0 (38.5/39.7)	b
	MAH 2	9	0.4 (0.3/1.2)		3.2 (2.7/4.1)		1.92 (0.97/2.92)		38.8 (38.4/39.5)	a
28-31	CG	20	0.5 (0.2/0.8)		3.7 (3.1/4.2)		1.92 (0.97/2.74)		38.9 (38.6/39.5)	
	MAP	23	0.5 (0.3/1.1)		3.7 (3.3/4.6)		1.99 (1.43/2.42)		39.1 (38.5/39.5)	
	MAH 2	9	0.5 (0.4/0.6)		3.5 (3.1/3.9)		2.17 (1.18/2.85)		38.9 (38.2/39.2)	
32-35	CG	20	0.5 (0.3/0.7)		3.7 (3.2/4.3)		2.14 (1.23/2.77)		38.8 (38.5/39.3)	
	MAP	23	0.6 (0.4/0.9)		3.5 (3.1/4.0)		2.06 (1.21/2.62)		38.8 (38.5/39.3)	
	MAH 2	9	0.5 (0.3/0.9)		3.8 (3.6/3.9)		1.91(1.55/3.44)		38.8 (38.3/39.3)	
36-39	CG	15	0.5 (0.3/1.3)		3.8 (3.2/4.3)		2.34 (1.74/2.74)		38.8 (38.4/39.4)	
	MAP	18	0.7 (0.3/1.2)		3.7 (3.3/4.3)		2.26 (1.94/2.82)		39.0 (38.6/39.7)	
	MAH 2	9	0.4 (0.3/0.5)		3.8 (3.7/4.0)		2.08 (1.75/3.41)		38.9 (38.4/39.3)	
40-43	CG	17	0.5 (0.4/1.2)		3.8 (3.4/4.4)		2.08 (1.44/2.96)		38.5 (38.2/39.0)	
	MAP	17	0.6 (0.3/0.9)		3.8 (3.5/4.1)		2.10 (1.49/2.70)		39.0 (38.3/39.8)	
	MAH 2	9	0.4 (0.3/0.6)		3.7 (3.5/4.2)		2.11 (1.80/2.87)		38.9 (38.7/39.6)	
44-47	CG	17	0.4 (0.3/2.2)		3.7 (3.5/4.1)		2.20 (0.73/3.07)		38.7 (38.4/39.2)	
	MAP	17	0.5 (0.3/1.2)		3.7 (3.4/4.1)		2.14 (1.20/2.85)		38.8 (38.4/39.6)	
	MAH 2	9	0.4 (0.3/2.6)		3.7 (3.4/3.9)		2.32 (1.49/2.92)		38.6 (38.1/39.0)	
48-51	CG	17	0.4 (0.3/0.7)		3.5 (3.3/4.0)		2.07 (1.53/2.91)		38.6 (38.3/39.3)	
	MAP	18	0.6 (0.2/1.3)		3.6 (3.2/4.1)		2.17 (1.43/2.83)		38.7 (38.2/39.3)	
	MAH 2	8	0.5 (0.2/1.9)		3.7 (3.5/4.4)		2.09 (1.45/3.30)		38.6 (38.4/38.8)	

wpi, week post-inoculation. CG, control group. MAP, group infected with *Mycobacterium avium* subsp. *paratuberculosis*. MAH 1, sub-group infected with *Mycobacterium avium* subsp. *hominissuis* with acute, severe form of infection. MAH 2, sub-group with chronic form of infection. Different letters indicate significant differences between groups within one period (Mann-Whitney *U*-test, $P < 0.05$). n.s., no significant differences between groups in the given period. From 28th week onwards Mann-Whitney *U*-test was not performed due to reduced numbers of observations. Significant differences within groups (Friedman test, $P < 0.05$) from 1st-3rd to 24th-27th wpi are given in S3, S4, S5 and S10 Tables.
