# Supplementary material for: Acid-base variables in acute and chronic form of nontuberculous mycobacterial infection in growing goats experimentally inoculated with Mycobacterium avium subsp. hominissuis or Mycobacterium avium subsp. paratuberculosis
Source: PLoS One. 2020 Dec 14;15(12):e0243892. doi: 10.1371/journal.pone.0243892 (PMC7735625; doi:10.1371/journal.pone.0243892)
Supplement: S3 Table — Additional information to S3 Table: P-values > 0.05 were considered not significant. (PDF) [file pone.0243892.s004.pdf]

**S3 Tables: P-values of Friedman test and consequently followed post hoc Wilcoxon rank-sum test applied to controls (CG) from the 1st-3rd to the 24th-27th week post-inoculation (wpi).**

**S3 A:** CG [Gluc] (Friedman test: P = <0.001, P-values of Wilcoxon rank-sum test are given below)

| wpi | 1-3 | 4-7 | 8-11 | 12-15 | 16-19 | 20-23 |
|---|---|---|---|---|---|---|
| 4-7 | 0.001 | | | | | |
| 8-11 | <0.001 | 0.016 | | | | |
| 12-15 | <0.001 | <0.001 | <0.001 | | | |
| 16-19 | <0.001 | <0.001 | <0.001 | 0.570 | | |
| 20-23 | <0.001 | <0.001 | <0.001 | 0.007 | 0.028 | |
| 24-27 | <0.001 | <0.001 | <0.001 | 0.004 | <0.001 | 0.322 |

**S3 B:** CG [$Na^+$] (Friedman test: P <0.001, P-values of Wilcoxon rank-sum test are given below)

| wpi | 1-3 | 4-7 | 8-11 | 12-15 | 16-19 | 20-23 |
|---|---|---|---|---|---|---|
| 4-7 | 0.983 | | | | | |
| 8-11 | 0.114 | 0.180 | | | | |
| 12-15 | 0.724 | 0.567 | 0.134 | | | |
| 16-19 | 0.059 | 0.127 | 0.624 | 0.015 | | |
| 20-23 | 0.032 | 0.045 | 0.237 | 0.011 | 0.473 | |
| 24-27 | 0.002 | 0.003 | <0.001 | 0.010 | <0.001 | <0.001 |

**S3 C:** CG [$Cl^-$] (Friedman test: P < 0.001, P-values of Wilcoxon rank-sum test are given below)

| wpi | 1-3 | 4-7 | 8-11 | 12-15 | 16-19 | 20-23 |
|---|---|---|---|---|---|---|
| 4-7 | 0.064 | | | | | |
| 8-11 | 0.002 | 0.117 | | | | |
| 12-15 | 0.028 | <0.001 | <0.001 | | | |
| 16-19 | 0.821 | 0.044 | 0.002 | 0.015 | | |
| 20-23 | 0.066 | 0.441 | 0.300 | <0.001 | 0.029 | |
| 24-27 | <0.001 | <0.001 | <0.001 | 0.062 | 0.003 | <0.001 |

**S3 D:** CG [$K^+$] (Friedman test: P = 0.004, P-values of Wilcoxon rank-sum test are given below)

| wpi | 1-3 | 4-7 | 8-11 | 12-15 | 16-19 | 20-23 |
|---|---|---|---|---|---|---|
| 4-7 | <0.001 | | | | | |
| 8-11 | 0.958 | 0.005 | | | | |
| 12-15 | 0.728 | 0.001 | 0.676 | | | |
| 16-19 | 0.236 | 0.004 | 0.280 | 0.206 | | |
| 20-23 | 0.035 | 0.107 | 0.072 | 0.012 | 0.186 | |
| 24-27 | 0.055 | 0.026 | 0.140 | 0.036 | 0.337 | 0.589 |

**S3 E:** CG [$Ca^{2+}$] (Friedman test: P <0.001, P-values of Wilcoxon rank-sum test are given below)

| wpi | 1-3 | 20-23 | 8-11 | 12-15 | 16-19 | 20-23 |
|---|---|---|---|---|---|---|
| 4-7 | <0.001 | | | | | |
| 8-11 | <0.001 | 0.464 | | | | |
| 12-15 | 0.002 | 0.094 | 0.111 | | | |
| 16-19 | <0.001 | 0.097 | 0.415 | 0.466 | | |
| 20-23 | <0.001 | 0.961 | 0.577 | 0.044 | 0.073 | |
| 24-27 | <0.001 | 0.148 | 0.246 | 0.197 | 0.837 | 0.062 |

**S3 F:** CG [L-Lac] (Friedman test: P <0.001, P-values of Wilcoxon rank-sum test are given below)

| wpi | 1-3 | 4-7 | 8-11 | 12-15 | 16-19 | 20-23 |
|---|---|---|---|---|---|---|
| 4-7 | 0.009 | | | | | |
| 8-11 | 0.001 | 0.321 | | | | |
| 12-15 | <0.001 | 0.002 | <0.001 | | | |
| 16-19 | <0.001 | <0.001 | 0.005 | 0.569 | | |
| 20-23 | <0.001 | <0.001 | <0.001 | 0.480 | 0.680 | |
| 24-27 | <0.001 | 0.001 | 0.004 | 0.793 | 0.751 | 0.913 |

**S3 G:** CG Hct (Friedman test: P <0.001, P-values of Wilcoxon rank-sum test are given below)

| wpi | 1-3 | 4-7 | 8-11 | 12-15 | 16-19 | 20-23 |
|---|---|---|---|---|---|---|
| 4-7 | <0.001 | | | | | |
| 8-11 | 0.002 | 0.009 | | | | |
| 12-15 | 0.201 | <0.001 | <0.001 | | | |
| 16-19 | 0.002 | <0.001 | <0.001 | <0.001 | | |
| 20-23 | 0.019 | <0.001 | <0.001 | 0.065 | 0.018 | |
| 24-27 | 0.475 | <0.001 | 0.002 | 0.394 | <0.001 | 0.004 |

**S3 H:** CG [iP] (Friedman test: P <0.001, P-values of Wilcoxon rank-sum test are given below)

| wpi | 1-3 | 4-7 | 8-11 | 12-15 | 16-19 | 20-23 |
|---|---|---|---|---|---|---|
| 4-7 | 0.006 | | | | | |
| 8-11 | 0.001 | 0.280 | | | | |
| 12-15 | <0.001 | <0.001 | <0.001 | | | |
| 16-19 | <0.001 | <0.001 | <0.001 | 0.140 | | |
| 20-23 | <0.001 | <0.001 | <0.001 | 0.136 | 0.417 | |
| 24-27 | <0.001 | <0.001 | <0.001 | 0.212 | 0.506 | 0.235 |

**S3 I:** CG body temperature (Friedman test: P = <0.001, P-values of Wilcoxon rank-sum test are given below)

| wpi | 1-3 | 4-7 | 8-11 | 12-15 | 16-19 | 20-23 |
|---|---|---|---|---|---|---|
| 4-7 | 0.002 | | | | | |
| 8-11 | <0.001 | 0.041 | | | | |
| 12-15 | <0.001 | 0.347 | 0.692 | | | |
| 16-19 | <0.001 | 0.454 | 0.486 | 0.601 | | |
| 20-23 | 0.005 | 0.958 | 0.075 | 0.296 | 0.486 | |
| 24-27 | <0.001 | 0.019 | 0.431 | 0.314 | 0.084 | 0.045 |

**S3 J:** CG $pCO_2(v)_{BT}$ (Friedman test: P <0.001, P-values of Wilcoxon rank-sum test are given below)

| wpi | 1-3 | 4-7 | 8-11 | 12-15 | 16-19 | 20-23 |
|---|---|---|---|---|---|---|
| 4-7 | <0.001 | | | | | |
| 8-11 | <0.001 | 0.085 | | | | |
| 12-15 | <0.001 | 0.123 | 0.758 | | | |
| 16-19 | <0.001 | 0.020 | 0.260 | 0.235 | | |
| 20-23 | <0.001 | 0.064 | 0.475 | 0.692 | 0.715 | |
| 24-27 | <0.001 | 0.019 | 0.049 | 0.186 | 0.749 | 0.523 |

**S3 K:** CG [$HCO_3^-$] (Friedman test: P = 0.001, P-values of Wilcoxon rank-sum test are given below)

| wpi | 1-3 | 4-7 | 8-11 | 12-15 | 16-19 | 20-23 |
|---|---|---|---|---|---|---|
| 4-7 | 0.045 | | | | | |
| 8-11 | 0.003 | 0.009 | | | | |
| 12-15 | <0.001 | <0.001 | <0.001 | | | |
| 16-19 | <0.001 | <0.001 | 0.001 | 0.784 | | |
| 20-23 | <0.001 | <0.001 | 0.042 | 0.038 | 0.005 | |
| 24-27 | <0.001 | 0.005 | 0.048 | 0.007 | 0.006 | 0.107 |

**S3 L:** CG [$HCO_3^-$(st)] (Friedman test: P < 0.001, P-values of Wilcoxon rank-sum test are given below)

| wpi | 1-3 | 4-7 | 8-11 | 12-15 | 16-19 | 20-23 |
|---|---|---|---|---|---|---|
| 4-7 | 0.603 | | | | | |
| 8-11 | 0.420 | 0.041 | | | | |
| 12-15 | <0.001 | <0.001 | <0.001 | | | |
| 16-19 | <0.001 | <0.001 | <0.001 | 0.681 | | |
| 20-23 | <0.001 | <0.001 | 0.036 | 0.019 | 0.006 | |
| 24-27 | 0.03 | 0.026 | 0.078 | 0.003 | 0.005 | 0.094 |

**S3 M:** CG [BE] (Friedman test: P <0.001, P-values of Wilcoxon rank-sum test are given below)

| wpi | 1-3 | 4-7 | 8-11 | 12-15 | 16-19 | 20-23 |
|---|---|---|---|---|---|---|
| **4-7** | 0.637 | | | | | |
| **8-11** | 0.287 | 0.031 | | | | |
| **12-15** | <0.001 | <0.001 | <0.001 | | | |
| **16-19** | <0.001 | <0.001 | <0.001 | 0.703 | | |
| **20-23** | <0.001 | <0.001 | 0.031 | 0.027 | 0.003 | |
| **24-27** | 0.006 | 0.012 | 0.061 | 0.003 | 0.004 | 0.115 |

**S3 N:** CG [$BE_{Ecf}$] (Friedman test: P <0.001, P-values of Wilcoxon rank-sum test are given below)

| wpi | 1-3 | 4-7 | 8-11 | 12-15 | 16-19 | 20-23 |
|---|---|---|---|---|---|---|
| **4-7** | 0.958 | | | | | |
| **8-11** | 0.136 | 0.014 | | | | |
| **12-15** | <0.001 | <0.001 | <0.001 | | | |
| **16-19** | <0.001 | <0.001 | <0.001 | 0.784 | | |
| **20-23** | <0.001 | <0.001 | 0.021 | 0.029 | 0.004 | |
| **24-27** | 0.002 | 0.007 | 0.046 | 0.003 | 0.006 | 0.091 |

**S3 O:** CG AG (Friedman test: P <0.001, P-values of Wilcoxon rank-sum test are given below)

| wpi | 1-3 | 4-7 | 8-11 | 12-15 | 16-19 | 20-23 |
|---|---|---|---|---|---|---|
| **4-7** | 0.073 | | | | | |
| **8-11** | 0.003 | 0.015 | | | | |
| **12-15** | <0.001 | <0.001 | 0.301 | | | |
| **16-19** | <0.001 | <0.001 | 0.293 | 0.681 | | |
| **20-23** | <0.001 | 0.002 | 0.345 | 0.485 | 0.858 | |
| **24-27** | <0.001 | 0.005 | 0.581 | 0.353 | 0.217 | 0.139 |

**S3 P:** CG $pH(v)_{BT}$ (Friedman test: P <0.001, P-values of Wilcoxon rank-sum test are given below)

| wpi | 1-3 | 4-7 | 8-11 | 12-15 | 16-19 | 20-23 |
|---|---|---|---|---|---|---|
| **4-7** | <0.001 | | | | | |
| **8-11** | 0.009 | 0.224 | | | | |
| **12-15** | 0.229 | <0.001 | <0.001 | | | |
| **16-19** | 0.447 | <0.001 | <0.001 | 0.702 | | |
| **20-23** | 0.260 | <0.001 | 0.007 | 0.010 | 0.002 | |
| **24-27** | 0.049 | 0.064 | 0.128 | <0.001 | 0.002 | 0.046 |

**S3 Q:** CG [TP] (Friedman test: P <0.001, P-values of Wilcoxon rank-sum test are given below)

| wpi | 1-3 | 4-7 | 8-11 | 12-15 | 16-19 | 20-23 |
|---|---|---|---|---|---|---|
| **4-7** | <0.001 | | | | | |
| **8-11** | <0.001 | 0.007 | | | | |
| **12-15** | <0.001 | 0.003 | 0.062 | | | |
| **16-19** | <0.001 | 0.002 | 0.189 | 0.346 | | |
| **20-23** | <0.001 | <0.001 | 0.052 | 0.891 | 0.648 | |
| **24-27** | <0.001 | 0.002 | 0.236 | 0.465 | 0.831 | 0.594 |

**S3 R:** CG [Alb] (Friedman test: P <0.001, P-values of Wilcoxon rank-sum test are given below)

| wpi | 1-3 | 4-7 | 8-11 | 12-15 | 16-19 | 20-23 |
|---|---|---|---|---|---|---|
| **4-7** | 0.001 | | | | | |
| **8-11** | <0.001 | <0.001 | | | | |
| **12-15** | <0.001 | 0.002 | 0.537 | | | |
| **16-19** | <0.001 | <0.001 | 0.161 | 0.581 | | |
| **20-23** | <0.001 | <0.001 | 0.248 | 0.659 | 0.935 | |
| **24-27** | <0.001 | <0.001 | 0.315 | 0.761 | 0.808 | 0.846 |

**S3 S:** CG [Gamma glob] (Friedman test: P <0.001, P-values of Wilcoxon rank-sum test are given below)

| wpi | 1-3 | 4-7 | 8-11 | 12-15 | 16-19 | 20-23 |
|---|---|---|---|---|---|---|
| **4-7** | <0.001 | | | | | |
| **8-11** | <0.001 | 0.094 | | | | |
| **12-15** | <0.001 | 0.091 | 0.267 | | | |
| **16-19** | <0.001 | 0.061 | 0.11 | 0.414 | | |
| **20-23** | <0.001 | 0.04 | 0.191 | 0.951 | 0.550 | |
| **24-27** | <0.001 | 0.14 | 0.315 | 0.465 | 0.181 | 0.098 |

**S3 T:** CG [Alpha 2] (Friedman test: P < 0.001, P-values of Wilcoxon rank-sum test are given below)

| wpi | 1-3 | 4-7 | 8-11 | 12-15 | 16-19 | 20-23 |
|---|---|---|---|---|---|---|
| **4-7** | <0.001 | | | | | |
| **8-11** | <0.001 | 0.113 | | | | |
| **12-15** | 0.002 | 0.889 | 0.079 | | | |
| **16-19** | <0.001 | 0.489 | 0.282 | 0.688 | | |
| **20-23** | 0.012 | 0.444 | 0.115 | 0.626 | 0.428 | |
| **24-27** | <0.001 | 0.845 | 0.139 | 0.741 | 0.626 | 0.888 |

**S3 U:** CG [Beta 1] (Friedman test: P = 0.013, P-values of Wilcoxon rank-sum test are given below)

| wpi | 1-3 | 4-7 | 8-11 | 12-15 | 16-19 | 20-23 |
|---|---|---|---|---|---|---|
| **4-7** | 0.088 | | | | | |
| **8-11** | 0.843 | 0.026 | | | | |
| **12-15** | 0.573 | 0.118 | 0.548 | | | |
| **16-19** | 0.765 | 0.211 | 0.270 | 0.875 | | |
| **20-23** | 0.006 | 0.654 | 0.027 | 0.009 | 0.045 | |
| **24-27** | 0.006 | 0.703 | 0.042 | 0.022 | 0.073 | 0.649 |

**S3 V:** CG [Beta 2] (Friedman test: P <0.001, P-values of Wilcoxon rank-sum test are given below)

| wpi | 1-3 | 4-7 | 8-11 | 12-15 | 16-19 | 20-23 |
|---|---|---|---|---|---|---|
| **4-7** | <0.001 | | | | | |
| **8-11** | <0.001 | 0.121 | | | | |
| **12-15** | <0.001 | 0.064 | 0.122 | | | |
| **16-19** | <0.001 | 0.767 | 0.041 | 0.007 | | |
| **20-23** | <0.001 | 0.503 | 0.121 | 0.135 | 0.601 | |
| **24-27** | <0.001 | 0.779 | 0.118 | 0.066 | 0.399 | 0.464 |

**S3 W:** CG $A_{tot\ TP}$ (Friedman test: P <0.001, P-values of Wilcoxon rank-sum test are given below)

| wpi | 1-3 | 4-7 | 8-11 | 12-15 | 16-19 | 20-23 |
|---|---|---|---|---|---|---|
| **4-7** | <0.001 | | | | | |
| **8-11** | <0.001 | 0.009 | | | | |
| **12-15** | <0.001 | 0.004 | 0.065 | | | |
| **16-19** | <0.001 | 0.002 | 0.205 | 0.338 | | |
| **20-23** | <0.001 | <0.001 | 0.049 | 0.855 | 0.626 | |
| **24-27** | <0.001 | 0.002 | 0.229 | 0.526 | 0.832 | 0.537 |

**S3 X:** CG $A_{tot\ Alb}$ (Friedman test: P <0.001, P-values of Wilcoxon rank-sum test are given below)

| wpi | 1-3 | 4-7 | 8-11 | 12-15 | 16-19 | 20-23 |
|---|---|---|---|---|---|---|
| **4-7** | <0.001 | | | | | |
| **8-11** | <0.001 | <0.001 | | | | |
| **12-15** | <0.001 | 0.002 | 0.559 | | | |
| **16-19** | <0.001 | <0.001 | 0.153 | 0.548 | | |
| **20-23** | <0.001 | <0.001 | 0.284 | 0.670 | 0.986 | |
| **24-27** | <0.001 | <0.001 | 0.33 | 0.684 | 0.833 | 0.961 |

**S3 Y:** CG $SID_{m3}$ (Friedman test: P = 0.001, P-values of Wilcoxon rank-sum test are given below)

| wpi | 1-3 | 4-7 | 8-11 | 12-15 | 16-19 | 20-23 |
|---|---|---|---|---|---|---|
| **4-7** | 0.455 | | | | | |
| **8-11** | 0.053 | 0.196 | | | | |
| **12-15** | 0.042 | 0.004 | <0.001 | | | |
| **16-19** | 0.037 | 0.008 | <0.001 | 0.703 | | |
| **20-23** | 0.573 | 0.548 | 0.076 | 0.104 | 0.032 | |
| **24-27** | 1.000 | 0.456 | 0.149 | 0.181 | 0.094 | 0.648 |

**S3 Z:** CG $SID_{m4}$ (Friedman test: P = <0.001, P-values of Wilcoxon rank-sum test are given below)

| wpi | 1-3 | 4-7 | 8-11 | 12-15 | 16-19 | 20-23 |
|---|---|---|---|---|---|---|
| **4-7** | 0.041 | | | | | |
| **8-11** | <0.001 | 0.107 | | | | |
| **12-15** | 0.338 | 0.011 | 0.002 | | | |
| **16-19** | 0.721 | 0.023 | 0.002 | 0.616 | | |
| **20-23** | 0.094 | 0.858 | 0.354 | 0.108 | 0.034 | |
| **24-27** | 0.323 | 0.595 | 0.199 | 0.128 | 0.097 | 0.397 |

**S3 AA:** CG $SID_{m5}$ (Friedman test: P <0.001, P-values of Wilcoxon rank-sum test are given below)

| wpi | 1-3 | 4-7 | 8-11 | 12-15 | 16-19 | 20-23 |
|---|---|---|---|---|---|---|
| **4-7** | 0.048 | | | | | |
| **8-11** | <0.001 | 0.097 | | | | |
| **12-15** | 0.308 | 0.012 | 0.002 | | | |
| **16-19** | 0.603 | 0.026 | 0.002 | 0.659 | | |
| **20-23** | 0.103 | 0.808 | 0.420 | 0.115 | 0.035 | |
| **24-27** | 0.378 | 0.595 | 0.211 | 0.136 | 0.097 | 0.721 |

**S3 AB:** CG $SIG_{TP}$ (Friedman test: P = 0.017, P-values of Wilcoxon rank-sum test are given below)

| wpi | 1-3 | 4-7 | 8-11 | 12-15 | 16-19 | 20-23 |
|---|---|---|---|---|---|---|
| **4-7** | 0.011 | | | | | |
| **8-11** | 0.128 | 0.212 | | | | |
| **12-15** | 0.976 | 0.007 | 0.212 | | | |
| **16-19** | 0.891 | 0.007 | 0.236 | 0.563 | | |
| **20-23** | 1.000 | 0.014 | 0.465 | 0.715 | 0.715 | |
| **24-27** | 0.171 | 0.287 | 0.738 | 0.503 | 0.181 | 0.308 |

**Additional information to S3 Tables:** P-values > 0.05 were considered not significant.

CG [Alpha 1] (Friedman test: P = 0.075)

CG Alb/Glob (Friedman test: P = 0.546)

CG $SIG_{Alb}$ (Friedman test: P = 0.431)
