# Supplementary material for: Acid-base variables in acute and chronic form of nontuberculous mycobacterial infection in growing goats experimentally inoculated with Mycobacterium avium subsp. hominissuis or Mycobacterium avium subsp. paratuberculosis
Source: PLoS One. 2020 Dec 14;15(12):e0243892. doi: 10.1371/journal.pone.0243892 (PMC7735625; doi:10.1371/journal.pone.0243892)
Supplement: S4 Table — Additional information to S4 Table: P-values > 0.05 were considered not significant. (PDF) [file pone.0243892.s005.pdf]

S4 Tables: P-values of Friedman test and consequently followed post hoc Wilcoxon rank-sum test applied to group MAP from the 1st-3rd to the 24th-27th week post-inoculation (wpi).

S4 A: MAP [Gluc] (Friedman test: $P < 0.001$; P-values of Wilcoxon rank-sum test are given below)

wpi	1-3	4-7	8-11	12-15	16-19	20-23
4-7	<0.001					
8-11	<0.001	0.388				
12-15	<0.001	<0.001	<0.001			
16-19	<0.001	<0.001	<0.001	0.037		
20-23	<0.001	<0.001	<0.001	0.468	0.846	
24-27	<0.001	<0.001	<0.001	<0.001	<0.001	<0.001

S4 B: MAP [Na^+] (Friedman test: $P < 0.001$; P-values of Wilcoxon rank-sum test are given below)

wpi	1-3	4-7	8-11	12-15	16-19	20-23
4-7	0.011					
8-11	0.001	0.005				
12-15	0.530	0.573	<0.001			
16-19	0.012	0.223	0.182	0.002		
20-23	<0.001	<0.001	0.836	<0.001	0.004	
24-27	0.005	0.133	0.611	<0.001	0.419	0.047

S4 C: MAP [Cl^-] (Friedman test: $P < 0.001$; P-values of Wilcoxon rank-sum test are given below)

wpi	1-3	4-7	8-11	12-15	16-19	20-23
4-7	0.031					
8-11	<0.001	0.014				
12-15	0.165	0.471	0.003			
16-19	0.716	0.021	<0.001	0.214		
20-23	0.001	0.077	0.191	0.032	0.009	
24-27	0.006	0.033	0.235	0.237	<0.001	0.700

S4 D: MAP [K^+] (Friedman test: $P < 0.001$; P-values of Wilcoxon rank-sum test are given below)

wpi	1-3	4-7	8-11	12-15	16-19	20-23
4-7	<0.001					
8-11	<0.001	0.482				
12-15	0.013	0.124	0.145			
16-19	0.015	0.483	0.551	0.456		
20-23	<0.001	0.754	0.434	0.021	0.054	
24-27	<0.001	0.389	0.206	0.003	0.006	0.600

S4 E: MAP [Ca^{2+}] (Friedman test: $P < 0.001$; P-values of Wilcoxon rank-sum test are given below)

wpi	1-3	4-7	8-11	12-15	16-19	20-23
4-7	<0.001					
8-11	<0.001	0.084				
12-15	<0.001	0.149	0.655			
16-19	<0.001	0.029	0.883	0.879		
20-23	<0.001	<0.001	0.037	0.016	0.034	
24-27	<0.001	0.002	0.039	0.016	0.037	0.584

S4 F: MAP [$_L$-Lac] (Friedman test: $P < 0.001$; P-values of Wilcoxon rank-sum test are given below)

wpi	1-3	4-7	8-11	12-15	16-19	20-23
4-7	<0.001					
8-11	<0.001	0.048				
12-15	<0.001	<0.001	<0.001			
16-19	<0.001	<0.001	<0.001	0.437		
20-23	<0.001	0.002	<0.001	0.238	0.059	
24-27	<0.001	<0.001	<0.001	0.109	0.334	<0.001

S4 G: MAP Hct (Friedman test: $P < 0.001$; P-values of Wilcoxon rank-sum test are given below)

wpi	1-3	4-7	8-11	12-15	16-19	20-23
4-7	<0.001					
8-11	<0.001	<0.001				
12-15	0.001	<0.001	<0.001			
16-19	0.003	<0.001	<0.001	<0.001		
20-23	0.002	<0.001	<0.001	<0.001	0.986	
24-27	0.182	<0.001	<0.001	<0.001	0.027	0.007

S4 H: MAP [iP] (Friedman test: $P < 0.001$; P-values of Wilcoxon rank-sum test are given below)

wpi	1-3	4-7	8-11	12-15	16-19	20-23
4-7	<0.001					
8-11	<0.001	0.904				
12-15	<0.001	<0.001	<0.001			
16-19	<0.001	<0.001	<0.001	0.013		
20-23	<0.001	<0.001	<0.001	0.001	0.614	
24-27	<0.001	<0.001	<0.001	0.003	0.661	0.573

S4 I: MAP body temperature (Friedman test: $P < 0.001$; P-values of Wilcoxon rank-sum test are given below)

wpi	1-3	4-7	8-11	12-15	16-19	20-23
4-7	0.476					
8-11	0.003	0.012				
12-15	0.006	0.004	0.064			
16-19	0.006	0.004	0.131	0.721		
20-23	<0.001	0.006	0.235	0.603	0.623	
24-27	<0.001	0.025	0.588	0.318	0.471	0.419

S4 J: MAP $pCO_2(v)_{BT}$ (Friedman test: $P < 0.001$; P-values of Wilcoxon rank-sum test are given below)

wpi	1-3	4-7	8-11	12-15	16-19	20-23
4-7	<0.001					
8-11	<0.001	0.138				
12-15	<0.001	0.003	0.245			
16-19	<0.001	0.010	0.437	0.620		
20-23	<0.001	0.033	0.891	0.248	0.406	
24-27	<0.001	0.193	0.543	0.055	0.143	0.367

S4 K: MAP [HCO_3^-] (Friedman test: $P < 0.001$ P-values of Wilcoxon rank-sum test are given below)

wpi	1-3	4-7	8-11	12-15	16-19	20-23
4-7	0.158					
8-11	<0.001	0.013				
12-15	<0.001	<0.001	<0.001			
16-19	<0.001	<0.001	<0.001	0.114		
20-23	<0.001	<0.001	0.002	0.602	0.179	
24-27	<0.001	<0.001	0.118	0.013	<0.001	<0.001

S4 L: MAP [HCO_3^-(st)] (Friedman test: $P < 0.001$; P values of Wilcoxon rank-sum test are given below)

wpi	1-3	4-7	8-11	12-15	16-19	20-23
4-7	0.141					
8-11	0.183	0.028				
12-15	<0.001	<0.001	0.005			
16-19	<0.001	<0.001	<0.001	0.122		
20-23	<0.001	<0.001	<0.001	0.573	0.137	
24-27	0.004	<0.001	0.286	0.018	<0.001	<0.001

S4 M: MAP [BE] (Friedman test: $P < 0.001$; P-values of Wilcoxon rank-sum test are given below)

wpi	1-3	4-7	8-11	12-15	16-19	20-23
4-7	0.188					
8-11	0.168	0.023				
12-15	<0.001	<0.001	0.002			
16-19	<0.001	<0.001	<0.001	0.118		
20-23	<0.001	<0.001	0.001	0.543	0.161	
24-27	<0.001	<0.001	0.124	0.022	<0.001	<0.001

S4 N: MAP [BE_{Ecf}] (Friedman test: $P < 0.001$; P-values of Wilcoxon rank-sum test are given below)

wpi	1-3	4-7	8-11	12-15	16-19	20-23
4-7	0.441					
8-11	0.033	0.018				
12-15	<0.001	<0.001	<0.001			
16-19	<0.001	<0.001	<0.001	0.114		
20-23	<0.001	<0.001	0.001	0.467	0.155	
24-27	<0.001	<0.001	0.095	0.022	<0.001	<0.001

S4 O: MAP AG (Friedman test: $P < 0.001$; P-values of Wilcoxon rank-sum test are given below)

wpi	1-3	4-7	8-11	12-15	16-19	20-23
4-7	0.097					
8-11	<0.001	<0.001				
12-15	<0.001	<0.001	<0.001			
16-19	<0.001	<0.001	0.084	0.013		
20-23	<0.001	<0.001	0.079	0.004	0.851	
24-27	<0.001	<0.001	0.421	<0.001	0.235	0.118

S4 P: MAP $pH(v)_{BT}$ (Friedman test: $P < 0.001$; P-values of Wilcoxon rank-sum test are given below)

wpi	1-3	4-7	8-11	12-15	16-19	20-23
4-7	<0.001					
8-11	<0.001	0.274				
12-15	0.069	<0.001	0.013			
16-19	0.809	<0.001	<0.001	0.116		
20-23	0.379	<0.001	0.002	0.259	0.437	
24-27	0.001	0.002	0.085	0.289	<0.001	0.001

S4 Q: MAP [TP] (Friedman test: $P < 0.001$; P-values of Wilcoxon rank-sum test are given below)

wpi	1-3	4-7	8-11	12-15	16-19	20-23
4-7	<0.001					
8-11	<0.001	<0.001				
12-15	<0.001	<0.001	0.029			
16-19	<0.001	<0.001	<0.001	0.402		
20-23	<0.001	<0.001	<0.001	0.007	0.022	
24-27	<0.001	<0.001	<0.001	0.007	0.005	0.891

S4 R: MAP [Alb] (Friedman test: $P < 0.001$; P-values of Wilcoxon rank-sum test are given below)

wpi	1-3	4-7	8-11	12-15	16-19	20-23
4-7	<0.001					
8-11	<0.001	0.006				
12-15	<0.001	0.002	0.043			
16-19	<0.001	0.939	0.106	0.025		
20-23	<0.001	0.037	0.979	0.301	0.026	
24-27	<0.001	0.043	0.447	0.069	0.124	0.521

S4 S: MAP [Gamma glob] (Friedman test: $P < 0.001$; P-values of Wilcoxon rank-sum test are given below)

wpi	1-3	4-7	8-11	12-15	16-19	20-23
4-7	<0.001					
8-11	<0.001	0.010				
12-15	<0.001	0.002	0.043			
16-19	<0.001	<0.001	<0.001	0.004		
20-23	<0.001	<0.001	<0.001	<0.001	<0.001	
24-27	<0.001	<0.001	<0.001	<0.001	<0.001	0.016

S4 T: MAP [Alpha 1] (Friedman test: $P < 0.001$; P-values of Wilcoxon rank-sum test are given below)

wpi	1-3	4-7	8-11	12-15	16-19	20-23
4-7	0.849					
8-11	<0.001	0.037				
12-15	0.006	0.229	0.144			
16-19	<0.001	<0.001	0.012	<0.001		
20-23	<0.001	0.017	0.986	0.261	0.058	
24-27	<0.001	0.144	0.206	0.914	0.006	0.158

S4 U: MAP [Alpha 2] (Friedman test: $P < 0.001$; P-values of Wilcoxon rank-sum test are given below)

wpi	1-3	4-7	8-11	12-15	16-19	20-23
4-7	0.004					
8-11	<0.001	0.462				
12-15	0.023	0.326	0.242			
16-19	0.291	0.007	0.001	0.013		
20-23	0.587	0.004	<0.001	0.016	0.469	
24-27	0.189	0.033	0.004	0.079	0.033	0.017

S4 V: MAP [Beta 1] (Friedman test: $P < 0.001$; P-values of Wilcoxon rank-sum test are given below)

wpi	1-3	4-7	8-11	12-15	16-19	20-23
4-7	0.468					
8-11	0.063	0.851				
12-15	0.042	0.157	0.815			
16-19	<0.001	0.007	0.043	0.092		
20-23	<0.001	0.004	0.026	0.027	0.745	
24-27	<0.001	0.004	0.010	0.019	0.979	0.731

S4 W: MAP [Beta 2] (Friedman test: $P < 0.001$; P-values of Wilcoxon rank-sum test are given below)

wpi	1-3	4-7	8-11	12-15	16-19	20-23
4-7	<0.001					
8-11	<0.001	<0.001				
12-15	<0.001	<0.001	0.115			
16-19	<0.001	<0.001	0.009	0.249		
20-23	<0.001	<0.001	0.254	0.455	0.188	
24-27	<0.001	<0.001	0.245	0.962	0.117	0.641

S4 X: MAP Alb/Glob (Friedman test: $P <0.001$; P-values of Wilcoxon rank-sum test are given below)

wpi	1-3	4-7	8-11	12-15	16-19	20-23
4-7	0.667					
8-11	0.339	0.242				
12-15	0.292	0.108	0.527			
16-19	<0.001	<0.001	0.003	0.008		
20-23	<0.001	<0.001	0.002	0.004	0.326	
24-27	<0.001	<0.001	<0.001	0.001	0.642	0.326

S4 Y: MAP $A_{tot\ TP}$ (Friedman test: $P < 0.001$; P-values of Wilcoxon rank-sum test are given below)

wpi	1-3	4-7	8-11	12-15	16-19	20-23
**4-7**	<0.001					
**8-11**	<0.001	<0.001				
**12-15**	<0.001	<0.001	0.033			
**16-19**	<0.001	<0.001	<0.001	0.432		
**20-23**	<0.001	<0.001	<0.001	0.007	0.022	
**24-27**	<0.001	<0.001	<0.001	0.008	0.006	0.844

S4 Z: MAP $A_{tot\ Alb}$ (Friedman test: $P < 0.001$; P-values of Wilcoxon rank-sum test are given below)

wpi	1-3	4-7	8-11	12-15	16-19	20-23
**4-7**	<0.001					
**8-11**	<0.001	0.005				
**12-15**	<0.001	0.002	0.043			
**16-19**	<0.001	0.952	0.101	0.027		
**20-23**	<0.001	0.036	0.964	0.313	0.026	
**24-27**	<0.001	0.044	0.469	0.068	0.131	0.499

S4 AA: MAP SID_{m3} (Friedman test: $P = 0.001$; P-values of Wilcoxon rank-sum test are given below)

wpi	1-3	4-7	8-11	12-15	16-19	20-23
**4-7**	0.063					
**8-11**	0.270	0.707				
**12-15**	0.966	0.222	0.608			
**16-19**	<0.001	0.008	0.004	0.003		
**20-23**	0.001	0.102	0.033	0.015	0.231	
**24-27**	0.022	0.809	0.668	0.225	0.009	0.012

S4 AB: MAP SID_{m4} (Friedman test: $P = 0.009$; P-values of Wilcoxon rank-sum test are given below)

wpi	1-3	4-7	8-11	12-15	16-19	20-23
**4-7**	0.417					
**8-11**	0.388	0.964				
**12-15**	0.038	0.222	0.249			
**16-19**	0.124	0.016	0.007	0.005		
**20-23**	0.561	0.197	0.084	0.026	0.305	
**24-27**	0.053	0.427	0.629	0.329	0.006	0.006

S4 AC: MAP SID_{m5} (Friedman test: $P = 0.011$; P-values of Wilcoxon rank-sum test are given below)

wpi	1-3	4-7	8-11	12-15	16-19	20-23
**4-7**	0.567					
**8-11**	0.503	0.943				
**12-15**	0.048	0.215	0.245			
**16-19**	0.089	0.018	0.007	0.005		
**20-23**	0.388	0.189	0.075	0.022	0.334	
**24-27**	0.08	0.483	0.681	0.313	0.008	0.006

S4 AD: MAP SIG_{TP} (Friedman test: $P < 0.001$; P-values of Wilcoxon rank-sum test are given below)

wpi	1-3	4-7	8-11	12-15	16-19	20-23
**4-7**	<0.001					
**8-11**	<0.001	0.045				
**12-15**	<0.001	<0.001	0.002			
**16-19**	<0.001	<0.001	0.279	0.009		
**20-23**	<0.001	0.035	0.939	<0.001	0.360	
**24-27**	<0.001	0.228	0.169	<0.001	0.032	0.068

S4 AE: MAP SIG_{Alb} (Friedman test: $P < 0.001$; P-values of Wilcoxon rank-sum test are given below)

wpi	1-3	4-7	8-11	12-15	16-19	20-23
**4-7**	<0.001					
**8-11**	<0.001	0.017				
**12-15**	0.330	<0.001	0.001			
**16-19**	0.383	<0.001	0.001	0.964		
**20-23**	0.006	<0.001	0.037	0.045	0.145	
**24-27**	<0.001	0.002	0.351	0.004	0.022	0.083

Additional information to S4 Tables: P-values > 0.05 were considered not significant.
