# Supplementary material for: Acid-base variables in acute and chronic form of nontuberculous mycobacterial infection in growing goats experimentally inoculated with Mycobacterium avium subsp. hominissuis or Mycobacterium avium subsp. paratuberculosis
Source: PLoS One. 2020 Dec 14;15(12):e0243892. doi: 10.1371/journal.pone.0243892 (PMC7735625; doi:10.1371/journal.pone.0243892)
Supplement: S5 Table — Additional information to S5 Table: P-values > 0.05 were considered not significant. (PDF) [file pone.0243892.s006.pdf]

S5 Tables: P-values of Friedman test and consequently followed post hoc Wilcoxon rank-sum test applied to sub-group MAH 2 from the 1st-3rd to the 24th-27th week post-inoculation (wpi).

S5 A: MAH 2 [Gluc] (Friedman test: $P < 0.001$; P-values of Wilcoxon rank-sum test are given below)

wpi	1-3	4-7	8-11	12-15	16-19	20-23
**4-7**	0.012					
**8-11**	0.012	0.889				
**12-15**	0.012	0.674	0.046			
**16-19**	0.012	0.263	0.012	0.018		
**20-23**	0.036	0.575	0.575	0.674	0.779	
**24-27**	0.012	0.036	0.012	0.017	0.035	0.017

S5 B: MAH 2 [Cl^-] (Friedman test: $P = 0.007$; P-values of Wilcoxon rank-sum test are given below)

wpi	1-3	4-7	8-11	12-15	16-19	20-23
**4-7**	0.733					
**8-11**	0.442	0.527				
**12-15**	0.017	0.020	0.071			
**16-19**	0.159	0.041	0.551	0.229		
**20-23**	0.018	0.019	0.024	0.230	0.173	
**24-27**	0.027	0.291	0.246	0.553	1.000	0.126

S5 C: MAH 2 [K^+] (Friedman test: $P = 0.001$; P-values of Wilcoxon rank-sum test are given below)

wpi	1-3	4-7	8-11	12-15	16-19	20-23
**4-7**	0.018					
**8-11**	0.018	0.866				
**12-15**	0.028	0.012	0.018			
**16-19**	0.080	0.263	0.123	0.017		
**20-23**	0.049	0.932	0.623	0.012	0.398	
**24-27**	0.400	0.063	0.401	0.024	0.833	0.310

S5 D: MAH 2 [$_L$-Lac] (Friedman test: $P = 0.001$; P-values of Wilcoxon rank-sum test are given below)

wpi	1-3	4-7	8-11	12-15	16-19	20-23
**4-7**	0.050					
**8-11**	0.050	0.237				
**12-15**	0.018	0.670	0.017			
**16-19**	0.017	0.551	0.012	0.861		
**20-23**	0.017	0.944	0.043	0.196	0.066	
**24-27**	0.012	0.599	0.080	0.932	0.854	0.498

S5 E: MAH 2 Hct (Friedman test: $P = 0.01$; P-values of Wilcoxon rank-sum test are given below)

wpi	1-3	4-7	8-11	12-15	16-19	20-23
**4-7**	0.036					
**8-11**	0.069	0.208				
**12-15**	0.574	0.025	0.176			
**16-19**	0.058	0.093	0.401	0.043		
**20-23**	0.107	0.036	0.161	0.161	0.401	
**24-27**	0.161	0.012	0.161	0.575	0.093	0.018

S5 F: MAH 2 [iP] (Friedman test: $P < 0.001$; P-values of Wilcoxon rank-sum test are given below)

wpi	1-3	4-7	8-11	12-15	16-19	20-23
**4-7**	0.012					
**8-11**	0.018	0.484				
**12-15**	0.012	0.050	0.128			
**16-19**	0.012	0.036	0.069	0.575		
**20-23**	0.012	0.025	0.017	0.401	0.779	
**24-27**	0.012	0.036	0.012	0.327	1.000	0.779

S5 G: MAH 2 body temperature (Friedman test: $P < 0.001$; P-values of Wilcoxon rank-sum test are given below)

wpi	1-3	4-7	8-11	12-15	16-19	20-23
**4-7**	0.498					
**8-11**	0.018	0.011				
**12-15**	0.008	0.008	0.031			
**16-19**	0.033	0.013	0.734	0.207		
**20-23**	0.008	0.008	0.018	0.232	0.049	
**24-27**	0.008	0.008	0.012	0.439	0.021	0.722

S5 H: MAH 2 $pCO_2(v)_{BT}$ (Friedman test: $P < 0.001$; P-values of Wilcoxon rank-sum test are given below)

wpi	1-3	4-7	8-11	12-15	16-19	20-23
**4-7**	0.575					
**8-11**	0.017	0.017				
**12-15**	0.012	0.012	0.263			
**16-19**	0.012	0.017	0.575	0.093		
**20-23**	0.017	0.025	1.000	0.263	0.674	
**24-27**	0.327	0.327	0.025	0.012	0.018	0.012

S5 I: MAH 2 [HCO_3^-] (Friedman test: $P < 0.001$; P-values of Wilcoxon rank-sum test are given below)

wpi	1-3	4-7	8-11	12-15	16-19	20-23
**4-7**	0.025					
**8-11**	1.000	0.208				
**12-15**	0.036	0.012	0.017			
**16-19**	0.012	0.012	0.025	0.161		
**20-23**	0.012	0.012	0.017	0.263	0.779	
**24-27**	0.123	0.017	0.401	0.069	0.012	0.017

S5 J: MAH 2 [$HCO_3^-(st)$] (Friedman test: $P < 0.001$; P-values of Wilcoxon rank-sum test are given below)

wpi	1-3	4-7	8-11	12-15	16-19	20-23
**4-7**	0.017					
**8-11**	0.263	0.483				
**12-15**	0.058	0.012	0.012			
**16-19**	0.012	0.012	0.025	0.093		
**20-23**	0.025	0.017	0.017	0.141	0.344	
**24-27**	0.091	0.012	0.183	0.161	0.012	0.025

S5 K: MAH 2 [BE] (Friedman test: $P = 0.001$; P-values of Wilcoxon rank-sum test are given below)

wpi	1-3	4-7	8-11	12-15	16-19	20-23
**4-7**	0.017					
**8-11**	0.263	0.484				
**12-15**	0.058	0.012	0.012			
**16-19**	0.012	0.012	0.017	0.092		
**20-23**	0.017	0.012	0.017	0.093	0.327	
**24-27**	0.106	0.012	0.161	0.208	0.012	0.017

S5 L: MAH 2 [BE_{Ecf}] (Friedman test: $P < 0.001$; P-values of Wilcoxon rank-sum test are given below)

wpi	1-3	4-7	8-11	12-15	16-19	20-23
**4-7**	0.025					
**8-11**	0.263	0.401				
**12-15**	0.050	0.012	0.012			
**16-19**	0.012	0.012	0.021	0.123		
**20-23**	0.017	0.012	0.017	0.183	0.484	
**24-27**	0.123	0.017	0.263	0.123	0.012	0.017

S5 M: MAH 2 AG (Friedman test: $P < 0.001$; P-values of Wilcoxon rank-sum test are given below)

wpi	1-3	4-7	8-11	12-15	16-19	20-23
4-7	0.036					
8-11	1.000	0.161				
12-15	0.012	0.012	0.012			
16-19	0.017	0.012	0.017	1.000		
20-23	0.866	0.208	0.674	0.093	0.161	
24-27	0.674	0.050	0.779	0.025	0.028	0.674

S5 N: MAH 2 $pH(v)_{BT}$ (Friedman test: $P < 0.001$; P-values of Wilcoxon rank-sum test are given below)

wpi	1-3	4-7	8-11	12-15	16-19	20-23
4-7	0.017					
8-11	0.025	0.161				
12-15	0.484	0.401	0.012			
16-19	0.123	0.017	0.017	0.068		
20-23	0.161	0.050	0.012	0.028	0.208	
24-27	0.889	0.123	0.017	0.779	0.035	0.025

S5 O: MAH 2 [TP] (Friedman test: $P < 0.001$; P-values of Wilcoxon rank-sum test are given below)

wpi	1-3	4-7	8-11	12-15	16-19	20-23
4-7	0.012					
8-11	0.012	0.025				
12-15	0.012	0.012	0.263			
16-19	0.012	0.012	0.092	0.624		
20-23	0.012	0.017	0.017	0.398	0.779	
24-27	0.012	0.012	0.05	0.141	0.401	0.484

S5 P: MAH 2 [Alb] (Friedman test: $P < 0.001$; P-values of Wilcoxon rank-sum test are given below)

wpi	1-3	4-7	8-11	12-15	16-19	20-23
4-7	0.036					
8-11	1.000	0.208				
12-15	0.018	0.012	0.043			
16-19	0.017	0.012	0.012	0.263		
20-23	0.012	0.012	0.012	0.093	0.293	
24-27	0.012	0.012	0.012	0.036	0.093	0.093

S5 Q: MAH 2 [Gamma glob] (Friedman test: $P < 0.001$; P-values of Wilcoxon rank-sum test are given below)

wpi	1-3	4-7	8-11	12-15	16-19	20-23
4-7	0.012					
8-11	0.012	0.012				
12-15	0.012	0.028	0.484			
16-19	0.012	0.036	0.263	0.484		
20-23	0.012	0.036	0.141	0.574	0.612	
24-27	0.012	0.093	0.069	0.042	0.050	0.069

S5 R: MAH 2 [Alpha 2] (Friedman test: $P < 0.001$; P-values of Wilcoxon rank-sum test are given below)

wpi	1-3	4-7	8-11	12-15	16-19	20-23
4-7	0.292					
8-11	0.866	0.309				
12-15	0.050	0.025	0.058			
16-19	0.091	0.018	0.028	0.889		
20-23	0.012	0.018	0.018	0.293	0.498	
24-27	0.018	0.012	0.012	0.149	0.463	0.400

S5 S: MAH 2 [Beta 2] (Friedman test: $P < 0.001$; P-values of Wilcoxon rank-sum test are given below)

wpi	1-3	4-7	8-11	12-15	16-19	20-23
4-7	0.008					
8-11	0.008	0.373				
12-15	0.008	0.123	0.674			
16-19	0.008	0.069	0.173	0.109		
20-23	0.012	0.015	0.008	0.008	0.091	
24-27	0.011	0.015	0.028	0.017	0.259	1.000

S5 T: MAH 2 Alb/Glob (Friedman test: $P < 0.001$; P-values of Wilcoxon rank-sum test are given below)

wpi	1-3	4-7	8-11	12-15	16-19	20-23
4-7	0.012					
8-11	0.012	0.735				
12-15	0.021	0.161	0.093			
16-19	0.069	0.012	0.012	0.208		
20-23	0.069	0.018	0.018	0.021	0.483	
24-27	0.160	0.012	0.012	0.012	0.028	0.011

S5 U: MAH 2 $A_{tot\ TP}$ (Friedman test: $P < 0.001$; P-values of Wilcoxon rank-sum test are given below)

wpi	1-3	4-7	8-11	12-15	16-19	20-23
4-7	0.012					
8-11	0.012	0.025				
12-15	0.012	0.012	0.262			
16-19	0.012	0.012	0.092	0.624		
20-23	0.012	0.017	0.017	0.398	0.779	
24-27	0.012	0.012	0.050	0.160	0.401	0.499

S5 V: MAH 2 $A_{tot\ Alb}$ (Friedman test: $P < 0.001$; P-values of Wilcoxon rank-sum test are given below)

wpi	1-3	4-7	8-11	12-15	16-19	20-23
4-7	0.035					
8-11	0.944	0.208				
12-15	0.018	0.012	0.046			
16-19	0.017	0.012	0.012	0.263		
20-23	0.012	0.012	0.012	0.063	0.327	
24-27	0.012	0.012	0.012	0.03	0.093	0.080

S5 W: MAH 2 SIG_{TP} (Friedman test: $P = 0.004$; P-values of Wilcoxon rank-sum test are given below)

wpi	1-3	4-7	8-11	12-15	16-19	20-23
4-7	0.012					
8-11	0.025	0.889				
12-15	0.208	0.050	0.017			
16-19	0.674	0.017	0.025	0.889		
20-23	0.093	0.889	0.779	0.093	0.093	
24-27	0.012	0.674	0.779	0.025	0.036	0.779

S5 X: MAH 2 SIG_{Alb} (Friedman test: $P = 0.007$; P-values of Wilcoxon rank-sum test are given below)

wpi	1-3	4-7	8-11	12-15	16-19	20-23
4-7	0.208					
8-11	0.612	0.779				
12-15	0.327	0.123	0.208			
16-19	0.327	0.182	0.327	0.726		
20-23	0.161	0.401	0.161	0.021	0.063	
24-27	0.012	0.012	0.025	0.012	0.018	0.674

Additional information to S5 Tables: P-values > 0.05 were considered not significant.

MAH 2 [Na^+] (Friedman test: P = 0.08)

MAH 2 [Ca^{2+}] (Friedman test: P = 0.08)

MAH 2 [Alpha 1] (Friedman test: P = 0.187

MAH 2 [Beta 1] P (Friedman test: P = 0.396)

MAH 2 SID_{m3} (Friedman test: P = 0.132)

MAH 2 SID_{m4} (Friedman test: P = 0.06)

MAH 2 SID_{m5} (Friedman test: P = 0.07)
