# Supplementary material for: Acid-base variables in acute and chronic form of nontuberculous mycobacterial infection in growing goats experimentally inoculated with Mycobacterium avium subsp. hominissuis or Mycobacterium avium subsp. paratuberculosis
Source: PLoS One. 2020 Dec 14;15(12):e0243892. doi: 10.1371/journal.pone.0243892 (PMC7735625; doi:10.1371/journal.pone.0243892)
Supplement: S6 Table — wpi, week post-inoculation. CG, control group. MAP, group infected with Mycobacterium avium subsp. paratuberculosis. MAH 1, sub-group infected with Mycobacterium avium subsp. hominissuis with acute, severe form of infection. MAH 2, sub-group with chronic form of infection. Different letters indicate significant differences between groups within one period (Mann-Whitney U-test, P < 0.05). n.s., no significant differences between groups in the given period. From 28th week onwards Mann-Whitney U-test was not performed due to reduced numbers of observations. Significant differences within groups (Friedman test, P < 0.05) from 1st-3rd to 24th-27th wpi are given in S3–S5, and S10 Tables. (PDF) [file pone.0243892.s007.pdf]

**S6 Table: Concentrations of total protein, albumin, gamma globulin in g/dL, and strong ion gap calculated on basis of total protein ($SIG_{TP}$) in mEq/L assessed in venous blood.**

| | | | [TP] g/dL | | [Alb] g/dL | | [Gamma glob] g/dl | | $SIG_{TP}$ mEq/L | |
|---|---|---|---|---|---|---|---|---|---|---|
| **wpi** | **group** | **n** | **median (min/max)** | | **median (min/max)** | | **median (min/max)** | | **median (min/max)** | |
| 1-3 | CG | 25 | 51.1 (45.3/58.0) | c | 30.4 (25.2/34.3) | b | 5.6 (2.6/12.4) | c | -1.87 (-6.29/2.01) | b |
| | MAP | 48 | 47.9 (43.6/53.9) | a | 28.6 (19.6/35.1) | a | 4.4 (1.5/12.3) | b | -3.41 (-6.01/1.16) | c |
| | MAH 2 | 9 | 50.2 (48.3/52.5) | bc | 28.8 (26.7/31.4) | ab | 3.9 (2.8/6.6) | ab | -4.95 (-7.90/-2.70) | a |
| | MAH 1 | 9 | 48.0 (42.1/53.1) | abc | 28.5 (24.9/34.1) | ab | 4.0 (2.4/6.7) | ab | -4.45 (-6.64/-2.26) | a |
| 4-7 | CG | 25 | 59.6 (47.0/72.8) | b | 34.5 (25.2/40.4) | b | 10.5 (4.6/18.9) | b | -0.18 (-5.68/3.97) | |
| | MAP | 48 | 56.4 (48.8/67.2) | a | 33.3 (19.9/40.8) | b | 7.6 (2.9/20.1) | a | -0.33 (-5.77/4.56) | n.s. |
| | MAH 2 | 9 | 55.1 (52.0/65.4) | ab | 25.1 (23.7/31.8) | a | 12.5 (6.6/21.1) | b | -0.78 (-5.00/1.54) | |
| | MAH 1 | 8 | 61.1 (46.1/78.0) | ab | 22.8 (14.8/30.7) | a | 16.9 (14.2/34.1) | c | 0.12 (-0.60/4.23) | |
| 8-11 | CG | 25 | 65.0 (54.6/73.3) | c | 37.6 (33.9/43.0) | c | 11.9 (7.8/17.7) | b | -1.30 (-3.82/3.12) | a |
| | MAP | 47 | 61.5 (52.5/76.7) | b | 36.4 (21.4/42.7) | c | 9.3 (6.2/32.4) | a | -1.09 (-6.17/6.75) | ab |
| | MAH 2 | 9 | 65.2 (54.1/71.7) | bc | 30.2 (19.5/37.9) | b | 19.4 (9.3/22.5) | c | -1.49 (-5.60/1.52) | ab |
| | MAH 1 | 6 | 33.5 (28.0/68.2) | a | 8.8 (6.0/20.1) | a | 10.85 (8.6/27.9) | abc | 0.60 (-5.39/4.39) | b |
| 12-15 | CG | 25 | 66.6 (56.9/76.7) | b | 38.9 (33.3/44.6) | b | 12.3 (9.1/18.9) | b | -1.49 (-7.56/2.41) | |
| | MAP | 47 | 64.1 (49.7/73.3) | a | 38.6 (21.0/42.7) | b | 10.2 (6.8/19.3) | a | -3.35 (-8.15/3.46) | n.s. |
| | MAH 2 | 9 | 67.6 (63.1/74.1) | ab | 35.2 (30.7/39.9) | a | 16.8 (9.9/20.9) | c | -5.02 (-6.41/-0.40) | |
| 16-19 | CG | 25 | 67.2 (56.8/75.9) | b | 39.3 (26.0/43.2) | b | 12.3 (9.7/17.5) | ab | -0.74 (-9.09/2.41) | ab |
| | MAP | 35 | 64.8 (56.6/76.9) | a | 35.3 (23.6/45.1) | a | 11.3 (7.1/17.5) | a | -1.43 (-7.70/2.18) | b |
| | MAH 2 | 9 | 66.0 (62.8/74.7) | ab | 37.0 (29.4/39.5) | a | 14.4 (10.1/22.8) | b | -3.97 (-6.98/-1.19) | a |
| 20-23 | CG | 23 | 66.6 (59.9/73.3) | | 39.2 (28.6/44,6) | b | 12.3 (10.4/17.5) | a | -1.22 (-5.66/3.75) | |
| | MAP | 34 | 67.6 (56.9/72.6) | n.s. | 37.6 (28.0/43.5) | a | 13.2 (7.6/19.3) | ab | -0.30 (-5.45/2.98) | n.s. |
| | MAH 2 | 9 | 68.0 (61.3/76.4) | | 37.6 (34.2/39.6) | ab | 15.8 (11.2/23.3) | b | -2.77 (-5.04/6.44) | |
| 24-27 | CG | 23 | 65.3 (60.5/75.6) | a | 39.0 (34.3/43.9) | b | 11.9 (9.6/17.0) | a | -1.03 (-4.17/1.94) | |
| | MAP | 34 | 66.3 (61.2/73.9) | a | 36.1 (29.8/41.4) | a | 13.7 (9.2/19.5) | b | -0.28 (-3.84/2.64) | n.s. |
| | MAH 2 | 9 | 70.1 (62.7/75.4) | b | 38.6 (36.1/43.0) | b | 14.0 (9.8/22.1) | b | -0.72 (-2.37/1.23) | |
| 28-31 | CG | 20 | 64.6 (59.0 /71.6) | | 37.4 (34.5/42.5) | | 12.0 (9.5/14.6) | | -0.22 (-1.55/3.23) | |
| | MAP | 23 | 66.4 (60.2/70.6) | | 36.4 (30.8/42.4) | | 14.1 (8.9/18.0) | | -0.42 (-4.66/2.02) | |
| | MAH 2 | 9 | 67.7 (64.2/77.8) | | 38.8 (34.6/42.9) | | 14.0 (10.9/21.3) | | 0.86 (-1.10/2.63) | |
| 32-35 | CG | 20 | 66.3 (62.3/71.9) | | 37.9 (35.7/41.8) | | 12.3 (9.9/17.5) | | -0.22 (-2.17/4.04) | |
| | MAP | 23 | 65.9 (62.2/70.9) | | 36.7 (31.4/41.3) | | 12.3 (10.4/18.7) | | -0.40 (-2.49/2.54) | |
| | MAH 2 | 9 | 67.1 (62.9/78.0) | | 38.1 (36.2/45.9) | | 12.6 (9.8/20.8) | | 0.30 (-0.80/3.06) | |
| 36-39 | CG | 15 | 65.8 (50.3/68.5) | | 37.2 (29.5/40.5) | | 11.6 (8.7/15.8) | | -0.07 (-1.75/0.99) | |
| | MAP | 18 | 66.3 (61.2/69.9) | | 36.4 (29.0/42.7) | | 13.0 (9.6/19.0) | | -0.42 (-3.28/2.81) | |
| | MAH 2 | 9 | 65.3 (61.2/72.7) | | 37.6 (31.6/40.4) | | 13.5 (11.6/19.2) | | 0.99 (-0.21/2.18) | |
| 40-43 | CG | 17 | 63.9 (57.6/70.1) | | 38.0 (32.1/42.8) | | 12.1 (8.7/14.0) | | 0.11 (-3.77/1.95) | |
| | MAP | 17 | 65.0 (60.7/81.4) | | 35.2 (28.6/40.7) | | 14.2 (10.5/25.5) | | -0.38 (-3.88/4.09) | |
| | MAH 2 | 9 | 66.5 (62.8/70.1) | | 35.6 (34.6/40.0) | | 12.8 (10.4/17.3) | | -2.70 (-4.02/-0.83) | |
| 44-47 | CG | 17 | 64.8 (57.7/69.4) | | 38.2 (31.7/42.5) | | 11.4 (9.4/14.2) | | -0.30 (-4.32/2.31) | |
| | MAP | 17 | 66.4 (60.2/75.3) | | 35.6 (29.4/40.0) | | 14.3 (11.2/22.4) | | -0.47 (-2.84/3.35) | |
| | MAH 2 | 9 | 67.5 (62.1/72.9) | | 37.9 (37.1/39.8) | | 12.9 (9.6/17.2) | | -1.92 (-2.73/-0.03) | |
| 48-51 | CG | 17 | 64.5 (57.3/73.6) | | 38.0 (31.9/41.4) | | 12.1 (9.1/15.3) | | -1.93 (-4.02/2.22) | |
| | MAP | 18 | 67.9 (61.4/77.0) | | 35.7 (29.3/42.8) | | 15.2 (11.2/22.9) | | 0.69 (-2.09/3.00) | |
| | MAH 2 | 8 | 67.7 (63.6/80.8) | | 36.9 (35.2/42.6) | | 14.3 (9.8/24.6) | | -0.98 (-2.49/2.57) | |

wpi, week post-inoculation. CG, control group. MAP, group infected with *Mycobacterium avium* subsp. *paratuberculosis*. MAH 1, sub-group infected with *Mycobacterium avium* subsp. *hominissuis* with acute, severe form of infection. MAH 2, sub-group with chronic form of infection. Different letters indicate significant differences between groups within one period (Mann-Whitney *U*-test, $P < 0.05$). n.s., no significant differences between groups in the given period. From 28$^{th}$ week onwards Mann-Whitney *U*-test was not performed due to reduced numbers of observations. Significant differences within groups (Friedman test, $P < 0.05$) from 1$^{st}$-3$^{rd}$ to 24$^{th}$-27$^{th}$ wpi are given in S3, S4, S5 and S10 Tables.
