# Supplementary material for: Acid-base variables in acute and chronic form of nontuberculous mycobacterial infection in growing goats experimentally inoculated with Mycobacterium avium subsp. hominissuis or Mycobacterium avium subsp. paratuberculosis
Source: PLoS One. 2020 Dec 14;15(12):e0243892. doi: 10.1371/journal.pone.0243892 (PMC7735625; doi:10.1371/journal.pone.0243892)
Supplement: S7 Table — wpi, week post-inoculation. CG, control group. MAP, group infected with Mycobacterium avium subsp. paratuberculosis. MAH 1, sub-group infected with Mycobacterium avium subsp. hominissuis with acute, severe form of infection. MAH 2, sub-group with chronic form of infection. Significant differences P < 0.05 calculated via Mann-Whitney U-test, a: CG/MAP, b: CG/MAH 2, c: CG/MAH 1, d: MAP/MAH 2, e: MAP/MAH 1, f: MAH 1/MAH 2. Significant differences P < 0.05 calculated via Friedman test from 1st-3rd to 24th-27th wpi, 1: within group CG, 2: within group MAP, 3: within sub-group MAH 2, 4: from 1st-3rd to 8th-11th wpi within sub-group MAH 1. Detailed P-values are given in S3–S5 and S10 Tables. (PDF) [file pone.0243892.s008.pdf]

S7 Table: Concentrations of sodium, chloride, potassium, and calcium in mmol/L assessed in venous blood.

			$[Na^+]$ mmol/L		$[Cl^-]$ mmol/L		$[K^+]$ mmol/L		$[Ca^{2+}]$ mmol/L	
**wpi**	**group**	**n**	**median (min/max)**		**median (min/max)**		**median (min/max)**		**median (min/max)**	
1-3	CG	25	140 (137/147)	a	102 (97/106)	a	4.3 (3.5/5.9)		1.34 (1.27/1.49)	
	MAP	48	143 (140/146)	b	103 (100/105)	b	4.6 (4.0/5.6)	n.s.	1.38 (1.27/1.52)	n.s.
	MAH 2	9	143 (141/145)	ab	103 (102/107)	bc	4.7 (4.0/5.2)		1.36 (1.28/1.45)	
	MAH 1	9	143 (141/147)	ab	105 (101/107)	c	4.5 (3.9/5)		1.38 (1.35/1.47)	
4-7	CG	25	140 (136/148)		100 (96/107)	a	4 (3.4/4.7)		1.27 (1.14/1.42)	a
	MAP	48	142 (137/148)	n.s.	102 (96/111)	b	4.1 (3.1/5.2)	n.s.	1.32 (1.10/1.44)	bc
	MAH 2	9	144 (142/146)		104 (102/108)	bc	4.0 (3.8/4.5)		1.32 (1.28/1.35)	c
	MAH 1	8	144 (141/145)		107 (104/109)	c	4.1 (3.6/4.6)		1.30 (1.23/1.35)	abc
8-11	CG	25	141 (137/147)	b	100 (95/104)	a	4.3 (3.6/5.2)		1.29 (1.18/1.39)	b
	MAP	47	139 (136/148)	ab	100 (95/108)	a	4.2 (3.2/5.4)	n.s.	1.29 (1.13/1.43)	bc
	MAH 2	9	144 (142/149)	c	104 (100/107)	b	4.1 (3.3/4.6)		1.29 (1.19/1.34)	bc
	MAH 1	6	138 (135/143)	a	105 (101/109)	b	4.0 (3.2/4.3)		1.06 (0.90/1.23)	a
12-15	CG	25	139 (137/150)	a	104 (98/108)	a	4.4 (3.7/5.6)	a	1.31 (1.20/1.40)	a
	MAP	47	143 (136/149)	ab	102 (94/110)	a	4.3 (3.5/5.6)	a	1.30 (1.11/1.43)	ab
	MAH 2	9	145 (144/148)	b	106 (104/109)	b	5.1 (4.5/6.0)	b	1.32 (1.30/1.37)	b
16-19	CG	25	139 (134/147)	a	100 (97/109)	a	4.3 (3.5/5.0)		1.29 (1.17/1.39)	a
	MAP	35	140 (134/148)	ab	103 (97/109)	a	4.2 (3.3/5.8)	n.s.	1.30 (1.14/1.45)	ab
	MAH 2	9	145 (142/146)	b	106 (102/108)	b	4.6 (3.7/4.7)		1.33 (1.26/1.42)	b
20-23	CG	23	140 (134/146)	a	100 (94/111)	a	4.1 (3.3/4.9)		1.27 (1.19/1.39)	a
	MAP	34	140 (136/145)	a	101 (97/109)	a	4.1 (3.3/5.0)	n.s.	1.27 (1.16/1.43)	a
	MAH 2	9	142 (141/147)	b	107 (104/112)	b	4.2 (3.7/4.7)		1.34 (1.21/1.38)	b
24-27	CG	23	144 (139/148)	b	104 (99/109)	b	4.3 (3.3/4.8)	ab	1.30 (1.23/1.35)	b
	MAP	34	140 (134/148)	a	102 (95/109)	a	4.1(3.5/5.3)	a	1.28 (1.17/1.36)	a
	MAH 2	9	145 (142/147)	b	105 (103/110)	b	4.3 (3.9/5.5)	b	1.31 (1.26/1.36)	b
28-31	CG	20	143 (141/146)		103 (99/106)		4.2 (3.7/5.8)		1.25 (1.16/1.32)	
	MAP	23	144 (143/147)		103 (100/106)		4.1 (3.3/4.7)		1.25 (1.18/1.36)	
	MAH 2	9	143 (142/145)		103 (100/108)		4.3 (4.0/5.2)		1.24 (1.12/1.30)	
32-35	CG	20	143 (141/146)		104 (101/108)		4.1 (3.7/5.2)		1.26 (1.21/1.32)	
	MAP	23	144 (142/145)		103 (100/108)		4.0 (3.6/4.8)		1.24 (1.17/1.38)	
	MAH 2	9	143 (142/146)		104 (102/105)		4.5 (3.8/5.0)		1.24 (1.16/1.27)	
36-39	CG	15	143 (140/145)		102 (100/107)		4.0 (3.6/5.6)		1.25 (1.17/1.33)	
	MAP	18	143 (141/145)		103 (100/107)		4.1 (3.3/4.7)		1.24 (1.12/1.33)	
	MAH 2	9	143 (142/144)		103 (101/105)		4.3 (3.8/4.4)		1.19 (1.08/1.20)	
40-43	CG	17	142 (140/147)		103 (99/106)		4.1 (3.6/5.8)		1.22 (1.17/1.27)	
	MAP	17	143 (142/146)		102 (100/105)		4.1 (3.4/4.6)		1.23 (1.12/1.26)	
	MAH 2	9	145 (145/146)		103 (102/107)		4.5 (3.9/4.7)		1.22 (1.11/1.24)	
44-47	CG	17	144 (140/148)		103 (101/107)		4.1 (3.6/5.6)		1.25 (1.16/1.31)	
	MAP	17	145 (139/148)		103 (101/106)		4.2 (3.7/5.0)		1.24 (1.19/1.32)	
	MAH 2	9	146 (144/148)		105 (104/107)		4.2 (3.8/4.5)		1.22 (1.12/1.35)	
48-51	CG	17	145 (143/150)		105 (100/108)		4.2 (3.8/4.7)		1.31 (1.21/1.37)	
	MAP	18	143 (141/146)		103 (99/108)		4.3 (3.4/4.7)		1.28 (1.13/1.34)	
	MAH 2	8	145 (143/147)		105 (102/108)		4.4 (3.7/4.8)		1.31 (1.14/1.33)	

wpi, week post-inoculation. CG, control group. MAP, group infected with *Mycobacterium avium* subsp. *paratuberculosis*. MAH 1, sub-group infected with *Mycobacterium avium* subsp. *hominissuis* with acute, severe form of infection. MAH 2, sub-group with chronic form of infection. Different letters indicate significant differences between groups within one period (Mann-Whitney *U*-test, $P < 0.05$). n.s., no significant differences between groups in the given period. From 28th week onwards Mann-Whitney *U*-test was not performed due to reduced numbers of observations. Significant differences within groups (Friedman test, $P < 0.05$) from 1st-3rd to 24th-27th wpi are given in S3, S4, S5 and S10 Tables.
