# Supplementary material for: Acid-base variables in acute and chronic form of nontuberculous mycobacterial infection in growing goats experimentally inoculated with Mycobacterium avium subsp. hominissuis or Mycobacterium avium subsp. paratuberculosis
Source: PLoS One. 2020 Dec 14;15(12):e0243892. doi: 10.1371/journal.pone.0243892 (PMC7735625; doi:10.1371/journal.pone.0243892)
Supplement: S8 Table — wpi, week post-inoculation. CG, control group. MAP, group infected with Mycobacterium avium subsp. paratuberculosis. MAH 1, sub-group infected with Mycobacterium avium subsp. hominissuis with acute, severe form of infection. MAH 2, sub-group with chronic form of infection. Different letters indicate significant differences between groups within one period (Mann-Whitney U-test, P < 0.05). n.s., no significant differences between groups in the given period. From 28th week onwards Mann-Whitney U-test was not performed due to reduced numbers of observations. Significant differences within groups (Friedman test, P < 0.05) from 1st-3rd to 24th-27th wpi are given in S3–S5, and S10 Tables. (PDF) [file pone.0243892.s009.pdf]

S8 Table: Concentrations of standard bicarbonate and standard base excess in mmol/L and hematocrit assessed in venous blood.

			$[HCO_3^-(st)]$ mmol/L		$[BE_{Ecf}]$ mmol/L		Hct %	
**wpi**	**group**	**n**	**median (min/max)**		**median(min/max)**		**median (min/max)**	
1-3	CG	25	28.4 (24.5/30.6)	b	5.9 (3.1/8.3)	b	35.3 (29.3/41.2)	
	MAP	48	28.1 (25.3/31.5)	b	5.8 (2.2/9.4)	b	34.9 (27.1/43.1)	n.s.
	MAH 2	9	26.2 (25.4/28.0)	a	2.9 (2.4/5.4)	a	35.8 (29.9/41.2)	
	MAH 1	9	26.9 (23.9/28.6)	a	3.7 (0.2/5.7)	a	34.6 (29.6/42.9)	
4-7	CG	25	28.1 (25.4/32.2)	b	5.5 (3.0/9.6)	b	40.1 (32.5/43.9)	b
	MAP	48	28.5 (23.3/33.5)	ab	5.8 (-0.6/11.7)	ab	40.5 (33.2/46.3)	b
	MAH 2	9	27.7 (25.0/31.0)	ab	4.9 (1.5/7.8)	ab	32.2 (20.9/36.1)	a
	MAH 1	8	26.0 (23.1/31.7)	a	2.7 (-0.7/8.7)	a	33.5 (21.4/35.5)	a
8-11	CG	25	27.6 (24.4/30.4)		4.5 (1.1/7.8)		37.4 (31.6/42.8)	bc
	MAP	47	27.3 (25.0/33.0)	n.s.	4.6 (1.3/10.8)	n.s.	38.2 (32.4/48.9)	c
	MAH 2	9	26.5 (23.1/30.3)		2.9 (-0.8/7.7)		31.8 (26.9/38.2)	ab
	MAH 1	6	27.8 (25.5/31.8)		3.8 (1.5/8.5)		31.7 (29.0/39.4)	a
12-15	CG	25	24.4 (20.6/27.7)	a	1.0 (-3.3/4.9)	a	34.4 (30.6/42.1)	a
	MAP	47	25.5 (19.7/30.6)	b	2.6 (-4.1/8.3)	b	36.1 (30.7/46.6)	b
	MAH 2	9	24.5 (22.2/28.6)	a	1.1 (-2.3/5.3)	a	33.7 (30.7/37.9)	a
16-19	CG	25	24.9 (21.2/28.3)	ab	1.6 (-3.0/5.8)	ab	32.5 (27.0/36.9)	
	MAP	35	24.8 (21.2/30.2)	b	1.4 (-3.1/7.4)	b	32.2 (24.6/38.7)	n.s.
	MAH 2	9	23.3 (22.4/24.9)	a	-0.8 (-1.4/1.2)	a	32.9 (27.8/34.6)	
20-23	CG	23	26.1 (21.1/28.9)	b	3.1 (-3.2/5.8)	b	33.2 (29.1/38.3)	
	MAP	34	25.9 (21.5/29.9)	b	2.7 (-2.9/7.4)	b	32.6 (26.2/39.6)	n.s.
	MAH 2	9	22.5 (20.9/27.2)	a	-1.4 (-3.7/3.8)	a	33.7 (28.4/37.0)	
24-27	CG	23	27.2 (20.8/30.8)	ab	4.1 (-3.6/8.0)	ab	34.7 (31.6/38.7)	
	MAP	34	27.1 (23.9/30.3)	b	4.1 (0.7/7.3)	b	34.0 (25.6/38.6)	n.s.
	MAH 2	9	25.5 (24.3/27.4)	a	2.5 (0.9/4.4)	a	34.1 (29.0/38.2)	
28-31	CG	20	28.0 (25.8/31.6)		5.0 (2.5/8.8)		36.1 (32.2/41.9)	
	MAP	23	28.1 (24.7/32.0)		5.3 (1.2/9.5)		35.2 (29.3/42.3)	
	MAH 2	9	27.8 (23.0/31.9)		4.6 (-0.8/9.2)		35.7 (30.1/38.5)	
32-35	CG	20	27.0 (25.7/29.7)		3.9 (2.4/7.0)		37.0 (31.5/47.9)	
	MAP	23	27.9 (24.4/29.7)		5.3 (1.0/7.0)		35.8 (29.6/40.6)	
	MAH 2	9	27.7 (25.9/28.7)		5.0 (2.6/6.2)		34.4 (31.4/40.3)	
36-39	CG	15	27.4 (25.0/31.3)		4.5 (1.9/9.2)		37.0 (31.9/41.5)	
	MAP	18	26.7 (23.5/30.3)		4.0 (0.1/7.6)		36.9 (32.6/40.4)	
	MAH 2	9	28.6 (26.7/28.8)		5.7 (3.5/6.0)		32.9 (30.7/33.6)	
40-43	CG	17	27.7 (24.5/30.1)		4.6 (1.8/7.6)		35.8 (31.1/40.4)	
	MAP	17	27.9 (24.5/29.4)		5.2 (1.0/6.4)		36.6 (31.0/40.9)	
	MAH 2	9	26.6 (25.3/28.3)		3.4 (2.0/5.4)		34.2 (31.7/38.5)	
44-47	CG	17	26.9 (25.0/30.2)		4.3 (1.7/7.5)		34.8 (30.5/40.7)	
	MAP	17	27.9 (25.7/29.5)		5.5 (2.6/6.6)		35.3 (29.7/40.7)	
	MAH 2	9	26.5 (25.9/27.5)		3.5 (2.6/4.6)		34.1 (32.4/41.4)	
48-51	CG	17	27.1 (25.0/32.2)		4.5 (2.1/9.7)		35.5 (31.3/40.8)	
	MAP	18	28.2 (23.9/30.3)		5.1 (0.1/7.9)		35.8 (27.3/42.7)	
	MAH 2	8	26.9 (23.2/28.6)		3.9 (-0.7/5.7)		35.2 (31.0/39.6)	

wpi, week post-inoculation. CG, control group. MAP, group infected with *Mycobacterium avium* subsp. *paratuberculosis*. MAH 1, sub-group infected with *Mycobacterium avium* subsp. *hominissuis* with acute, severe form of infection. MAH 2, sub-group with chronic form of infection. Different letters indicate significant differences between groups within one period (Mann-Whitney *U*-test, $P < 0.05$). n.s., no significant differences between groups in the given period. From 28th week onwards Mann-Whitney *U*-test was not performed due to reduced numbers of observations. Significant differences within groups (Friedman test, $P < 0.05$) from 1st-3rd to 24th-27th wpi are given in S3, S4, S5 and S10 Tables.
