# Supplementary material for: Acid-base variables in acute and chronic form of nontuberculous mycobacterial infection in growing goats experimentally inoculated with Mycobacterium avium subsp. hominissuis or Mycobacterium avium subsp. paratuberculosis
Source: PLoS One. 2020 Dec 14;15(12):e0243892. doi: 10.1371/journal.pone.0243892 (PMC7735625; doi:10.1371/journal.pone.0243892)
Supplement: S9 Table — wpi, week post-inoculation. CG, control group. MAP, group infected with Mycobacterium avium subsp. paratuberculosis. MAH 1, sub-group infected with Mycobacterium avium subsp. hominissuis with acute, severe form of infection. MAH 2, sub-group with chronic form of infection. Different letters indicate significant differences between groups within one period (Mann-Whitney U-test, P < 0.05). n.s., no significant differences between groups in the given period. From 28th week onwards Mann-Whitney U-test was not performed due to reduced numbers of observations. Significant differences within groups (Friedman test, P < 0.05) from 1st-3rd to 24th-27th wpi are given in S3–S5, and S10 Tables. (PDF) [file pone.0243892.s010.pdf]

S9 Table: Concentrations of beta 1, beta 2, alpha 1, and alpha 2 globulin in g/dL assessed in venous blood.

			[Beta 1] g/dL		[Beta 2] g/dL		[Alpha 1] g/dL		[Alpha 2] g/dL	
**wpi**	**group**	**n**	**median (min/max)**		**median (min/max)**		**median (min/max)**		**median (min/max)**	
1-3	CG	25	35.3 (29.3/41.2)		1.1 (0.5/1.9)	abc	1.3 (0.6/1.6)		5.9 (4.3/6.8)	a
	MAP	48	34.9 (27.1/43.1)	n.s.	1.0 (0.6/1.8)	a	1.4 (0.7/2.1)	n.s.	5.7 (3.3/7.7)	ab
	MAH 2	9	35.8 (29.9/41.2)		1.3 (0.7/1.6)	c	1.4 (0.7/2.0)		6.6 (5.3/8.1)	b
	MAH 1	9	34.6 (29.6/42.9)		1.1 (0.7/1.2)	ab	1.1 (0.7/1.8)		5.4 (3.8/8.4)	ab
4-7	CG	25	40.1 (32.5/43.9)	b	1.6 (1.0/3.0)	b	1.5 (0.7/2.1)	ab	5.0 (3.1/6.8)	a
	MAP	48	40.5 (33.2/46.3)	b	1.4 (0.7/2.5)	a	1.4 (0.6/3.6)	a	5.3 (2.3/8.6)	ab
	MAH 2	9	32.2 (20.9/36.1)	a	2.2 (1.6/3.8)	c	1.4 (1.0/2.2)	ab	7.0 (5.1/8.4)	c
	MAH 1	8	33.5 (21.4/35.5)	a	2.0 (1.0/4.6)	bc	1.9 (1.3/3.0)	b	6.7 (3.8/8.5)	bc
8-11	CG	25	37.4 (31.6/42.8)	bc	1.8 (1.0/2.4)	a	1.4 (0.8/2.3)		4.9 (2.8/7.2)	a
	MAP	47	38.2 (32.4/48.9)	c	1.9 (1.0/4.2)	ab	1.6 (0.8/2.6)	n.s.	5.4 (2.3/7.9)	ab
	MAH 2	9	31.8 (26.9/38.2)	ab	2.1 (1.5/3.7)	b	1.8 (1.3/2.0)		6.5 (5.1/7.8)	c
	MAH 1	6	31.7 (29.0/39.4)	a	1.4 (0.4/5.0)	ab	1.2 (1.0/2.2)		5.7 (3.8/7.0)	abc
12-15	CG	25	34.4 (30.6/42.1)	a	1.7 (1/3.7.0)		1.5 (0.9/4.3)		4.9 (3.0/7.2)	
	MAP	47	36.1 (30.7/46.6)	b	1.9 (1.0/9.5)	n.s.	1.5 (0.9/2.3)	n.s.	5.6 (2.4/8.1)	n.s.
	MAH 2	9	33.7 (30.7/37.9)	a	2.0 (1.3/3.0)		1.6 (1.1/1.8)		6.3 (4.3/7.2)	
16-19	CG	25	32.5 (27.0/36.9)		1.6 (1.0/2.5)	a	1.2 (0.8/3.0)	a	5.2 (2.6/7.0)	a
	MAP	35	32.2 (24.6/38.7)	n.s.	2.1 (1.2/10.4)	b	1.9 (1.0/3.9)	b	6.1 (2.7/8.7)	b
	MAH 2	9	32.9 (27.8/34.6)		1.8 (1.2/4.0)	ab	1.3 (1.1/1.9)	a	6.4 (3.9/7.0)	b
20-23	CG	23	33.2 (29.1/38.3)		1.6 (1.2/5.5)	a	1.5 (0.9/2.6)		5.4 (2.9/6.9)	
	MAP	34	32.6 (26.2/39.6)	n.s.	1.9 (1.4/6.9)	b	1.6 (1.0/2.9)	n.s.	5.8 (2.4/9.3)	n.s.
	MAH 2	9	33.7 (28.4/37.0)		1.9 (1.2/2.4)	ab	1.3 (1.1/1.9)		6.0 (4.2/6.9)	
24-27	CG	23	34.7 (31.6/38.7)		1.6 (0.9/2.8)	a	1.4 (1.0/2.0)		4.8 (3.2/6.5)	
	MAP	34	34.0 (25.6/38.6)	n.s.	1.9 (1.4/5.8)	b	1.5 (1.0/2.2)	n.s.	5.6 (2.8/8.0)	n.s.
	MAH 2	9	34.1 (29.0/38.2)		1.7 (0.9/2.6)	ab	1.3 (1.2/2.0)		6.0 (4.4/6.8)	
28-31	CG	20	36.1 (32.2/41.9)		1.6 (1.1/2.1)		1.4 (0.8/1.8)		5.2 (3.2/6.7)	
	MAP	23	35.2 (29.3/42.3)		1.8 (1.2/2.8)		1.8 (1.1/2.3)		5.6 (2.6/8.5)	
	MAH 2	9	35.7 (30.1/38.5)		1.4 (1.0/2.8)		1.4 (1.0/1.8)		5.7 (4.7/7.4)	
32-35	CG	20	37.0 (31.5/47.9)		1.8 (0.9/3.5)		1.6 (0.9/2.3)		5.4 (2.8/6.7)	
	MAP	23	35.8 (29.6/40.6)		1.8 (1.1/2.9)		1.5 (0.9/2.2)		5.3 (2.4/8.4)	
	MAH 2	9	34.4 (31.4/40.3)		1.7 (1.0/3.3)		1.5 (1.0/1.7)		5.9 (4.6/7.9)	
36-39	CG	15	37.0 (31.9/41.5)		1.6 (0.9/4.0)		1.3 (0.9/2.0)		5.1 (3.7/7.7)	
	MAP	18	36.9 (32.6/40.4)		1.9 (1.1/5.4)		1.7 (0.9/2.4)		5.7 (2.5/7.7)	
	MAH 2	9	32.9 (30.7/33.6)		2.3 (1.7/3.9)		1.5 (1.2/1.7)		5.6 (4.8/6.4)	
40-43	CG	17	35.8 (31.1/40.4)		1.6 (0.9/2.2)		1.2 (0.9/1.7)		4.6 (3.1/6.0)	
	MAP	17	36.6 (31.0/40.9)		1.9 (1.2/4.0)		1.6 (0.8/2.6)		5.1 (2.5/8.6)	
	MAH 2	9	34.2 (31.7/38.5)		1.5 (1.3/2.5)		1.3 (1.1/1.7)		5.3 (4.4/7.2)	
44-47	CG	17	34.8 (30.5/40.7)		1.4 (0.7/2.5)		1.2 (0.5/2.2)		4.9 (3.1/6.1)	
	MAP	17	35.3 (29.7/40.7)		1.5 (1.3/3.5)		1.6 (1.0/2.7)		5.2 (2.4/8.1)	
	MAH 2	9	34.1 (32.4/41.4)		1.4 (0.9/2.6)		1.3 (1.1/1.8)		5.5 (4.1/7.2)	
48-51	CG	17	35.5 (31.3/40.8)		1.3 (0.9/3.7)		1.5 (1.0/2.2)		5.0 (3.0/6.5)	
	MAP	18	35.8 (27.3/42.7)		1.5 (1.0/3.0)		1.5 (0.9/2.4)		5.3 (3.6/8.3)	
	MAH 2	8	35.2 (31.0/39.6)		1.4 (0.9/3.6)		1.6 (1.3/2.0)		6.0 (4.6/7.6)	

wpi, week post-inoculation. CG, control group. MAP, group infected with *Mycobacterium avium* subsp. *paratuberculosis*. MAH 1, sub-group infected with *Mycobacterium avium* subsp. *hominissuis* with acute, severe form of infection. MAH 2, sub-group with chronic form of infection. Different letters indicate significant differences between groups within one period (Mann-Whitney *U*-test, $P < 0.05$). n.s., no significant differences between groups in the given period. From 28th week onwards Mann-Whitney *U*-test was not performed due to reduced numbers of observations. Significant differences within groups (Friedman test, $P < 0.05$) from 1st-3rd to 24th-27th wpi are given in S3, S4, S5 and S10 Tables.
