# Supplementary material for: Acid-base variables in acute and chronic form of nontuberculous mycobacterial infection in growing goats experimentally inoculated with Mycobacterium avium subsp. hominissuis or Mycobacterium avium subsp. paratuberculosis
Source: PLoS One. 2020 Dec 14;15(12):e0243892. doi: 10.1371/journal.pone.0243892 (PMC7735625; doi:10.1371/journal.pone.0243892)
Supplement: S10 Table — Additional information to S10 Table: P-values > 0.05 were considered not significant. (PDF) [file pone.0243892.s011.pdf]

S10 Tables: P-values of Friedman test and consequently followed post hoc Wilcoxon rank-sum test applied to sub-group MAH 1 from the 1st-3rd to the 8th-11th week post-inoculation (wpi).

S6 A: MAH 1 [Gluc] (Friedman test: P = 0.009; P-values of Wilcoxon rank-sum test are given below)

wpi	1-3	4-7
4-7	0.028	
8-11	0.028	0.345

S6 B: MAH 1 [Na^+] (Friedman test: P = 0.032; P-values of Wilcoxon rank-sum test are given below)

wpi	1-3	4-7
4-7	0.68	
8-11	0.046	0.027

S6 C: MAH 1 [K^+] (Friedman test: P = 0.018; P-values of Wilcoxon rank-sum test are given below)

wpi	1-3	4-7
4-7	0.028	
8-11	0.043	0.463

S6 D: MAH 1 [Ca^{2+}] (Friedman test: P = 0.003; P-values of Wilcoxon rank-sum test are given below)

wpi	1-3	4-7
4-7	0.028	
8-11	0.028	0.028

S6 E: MAH 1 [$_L$-Lac] (Friedman test: P = 0.042; P-values of Wilcoxon rank-sum test are given below)

wpi	1-3	4-7
4-7	0.248	
8-11	0.027	0.917

S6 F: MAH 1 [iP] (Friedman test: P = 0.03; P-values of Wilcoxon rank-sum test are given below)

wpi	1-3	4-7
4-7	0.046	
8-11	0.028	0.917

S6 G: MAH 1 pCO_2 (Friedman test: P = 0.006; P-values of Wilcoxon rank-sum test are given below)

wpi	1-3	4-7
4-7	0.138	
8-11	0.028	0.028

S6 H: MAH 1 AG (Friedman test: P = 0.03; P-values of Wilcoxon rank-sum test are given below)

wpi	1-3	4-7
4-7	0.116	
8-11	0.028	0.046

S6 I: MAH 1 $pH(v)_{BT}$ (Friedman test: P = 0.009; P-values of Wilcoxon rank-sum test are given below)

wpi	1-3	4-7
4-7	0.116	
8-11	0.028	0.028

S6 J: MAH 1 [TP] (Friedman test: P = 0.04; P-values of Wilcoxon rank-sum test are given below)

wpi	1-3	4-7
4-7	0.028	
8-11	0.462	0.046

S6 K: MAH 1 [Alb] (Friedman test: P = 0.006; P-values of Wilcoxon rank-sum test are given below)

wpi	1-3	4-7
4-7	0.058	
8-11	0.028	0.028

S6 L: MAH 1 [Gamma glob] (Friedman test: P = 0.006; P-values of Wilcoxon rank-sum test are given below)

wpi	1-3	4-7
4-7	0.027	
8-11	0.028	0.345

S6 M: MAH 1 Alb/Glob (Friedman test: P = 0.002; P-values of Wilcoxon rank-sum test are given below)

wpi	1-3	4-7
4-7	0.028	
8-11	0.028	0.028

S6 N: MAH 1 $A_{tot\ TP}$ (Friedman test: P = 0.042; P-values of Wilcoxon rank-sum test are given below)

wpi	1-3	4-7
4-7	0.028	
8-11	0.463	0.046

S6 O: MAH 1 $A_{tot\ Alb}$ (Friedman test: P = 0.006; P-values of Wilcoxon rank-sum test are given below)

wpi	1-3	4-7
4-7	0.046	
8-11	0.028	0.028

S6 P: MAH 1 SID_{m3} (Friedman test: P = 0.03; P-values of Wilcoxon rank-sum test are given below)

wpi	1-3	4-7
4-7	0.173	
8-11	0.028	0.075

S6 Q: MAH 1 SID_{m4} (Friedman test: P = 0.03; P-values of Wilcoxon rank-sum test are given below)

wpi	1-3	4-7
4-7	0.141	
8-11	0.028	0.075

S6 R: MAH 1 SID_{m5} (Friedman test: P = 0.03; P-values of Wilcoxon rank-sum test are given below)

wpi	1-3	4-7
4-7	0.116	
8-11	0.028	0.074

S6 S: MAH 1 SIG_{TP} (Friedman test: P = 0.042; P-values of Wilcoxon rank-sum test are given below)

wpi	1-3	4-7
**4-7**	0.028	
**8-11**	0.046	0.753

Additional information to S6 Tables: P-values > 0.05 were considered not significant.

MAH 1 Hct (Friedman test: P = 0.51)
MAH 1 [HCO_3^-] (Friedman test: P = 0.51)
MAH 1 [BE] (Friedman test: P = 0.61)
MAH 1 body temp. (Friedman test: P = 0.2)
MAH 1 [Beta 1] (Friedman test: P = 0.87)
MAH 1 SIG_{Alb} (Friedman test: P = 0.85)
MAH 1 [Cl^-] (Friedman test: P = 0.28)
MAH 1 [HCO_3^-(st)] (Friedman test: P = 0.61)
MAH 1 [BE_{Ecf}] (Friedman test: P = 0.85)
MAH 1 [Alpha 1] (Friedman test: P = 0.112)
MAH 1 [Alpha 2] (Friedman test: P = 0.119)
MAH 1 [Beta 2] (Friedman test: P = 0.07)
